# Targeting VEGFR2 with Ramucirumab strongly impacts effector/ activated regulatory T cells and CD8^+^ T cells in the tumor microenvironment

**DOI:** 10.1186/s40425-018-0403-1

**Published:** 2018-10-11

**Authors:** Yasuko Tada, Yosuke Togashi, Daisuke Kotani, Takeshi Kuwata, Eichi Sato, Akihito Kawazoe, Toshihiko Doi, Hisashi Wada, Hiroyoshi Nishikawa, Kohei Shitara

**Affiliations:** 1 Division of Cancer Immunology, Research Institute / Exploratory Oncology Research and Clinical Trial Center (EPOC), National Cancer Center, 6-5-1 Kashiwanoha, Kashiwa, Chiba 277-8577 Japan; 2 Department of Gastroenterology and Gastrointestinal Oncology, National Cancer Center Hospital East, 6-5-1 Kashiwanoha, Kashiwa, Chiba 277-8577 Japan; 3 Department of Pathology and Clinical Laboratories, National Cancer Center Hospital East, Chiba, Japan; 4 Department of Pathology, Institute of Medical Science, Tokyo Medical University, Tokyo, Japan; 5 Department of Clinical Research in Tumor Immunology, Osaka University Graduate School of Medicine, Osaka, Japan; 6 Department of Immunology, Nagoya University Graduate School of Medicine, Nagoya, Japan

**Keywords:** Regulatory T cells, PD-1, Ramucirumab, VEGFR2, Gastric cancer

## Abstract

**Background:**

Several studies have established a correlation between the VEGF–VEGFR2 axis and an immunosuppressive microenvironment; this immunosuppression can be overcome by anti-angiogenic reagents, such as ramucirumab (RAM). However, little is known about the immunological impact of anti-angiogenic reagents within the tumor microenvironment in human clinical samples. This study aimed at investigating the effects of RAM on the tumor microenvironmental immune status in human cancers.

**Methods:**

We prospectively enrolled 20 patients with advanced gastric cancer (GC) who received RAM-containing chemotherapy. We obtained paired samples from peripheral blood mononuclear cells (PBMCs) and tumor-infiltrating lymphocytes (TILs) in primary tumors both pre- and post-RAM therapy to assess immune profiles by immunohistochemistry and flow cytometry.

**Results:**

Within the tumor microenvironment, both PD-L1 expression and CD8^+^ T-cell infiltration increased after RAM-containing therapies. In addition, CD45RA^−^FOXP3^high^CD4^+^ cells (effector regulatory T cells [eTreg cells]) and PD-1 expression by CD8^+^ T cells were significantly reduced in TILs compared with PBMCs after RAM-containing therapies. Patients with partial response and longer progression-free survival had significantly higher pre-treatment eTreg frequencies in TILs than those with progressive disease. In in vitro analysis, VEGFR2 was highly expressed by eTreg cells. Further, VEGFA promoted VEGFR2^+^ eTreg cell proliferation, and this effect could be inhibited by RAM.

**Conclusions:**

This study suggests that the frequency of eTreg cells in TILs could be a biomarker for stratifying clinical responses to RAM-containing therapies. Further, we propose that RAM may be employed as an immuno-modulator in combination with immune checkpoint blockade.

**Electronic supplementary material:**

The online version of this article (10.1186/s40425-018-0403-1) contains supplementary material, which is available to authorized users.

## Background

Recent advances in cancer immunotherapy, including the development of immune checkpoint blockades (ICBs), such as those targeting PD-1, PD-L1, and CTLA-4, have resulted in a paradigm shift in cancer treatment. In fact, the U.S. Food and Drug Administration has granted approvals for ICB use across several tumor types [1–9]. The capacity of tumor cells to cultivate immune escape mechanisms is one of the leading processes involved in tumor appearance and growth [10]; ICB is known to target one of these escape mechanisms. However, to date, the therapeutic efficacy of ICB remains insufficient, necessitating the establishment of more effective therapies, including combination therapies [1–9].

Ramucirumab (RAM), a fully humanized IgG1 monoclonal anti-vascular endothelial growth factor receptor 2 (VEGFR2) antibody, has been shown to exhibit significant efficacy as an anti-angiogenic agent in several cancers, including non-small cell lung cancer (NSCLC), gastric cancer (GC), colorectal cancer, and bladder cancer [11–15]. Further, several studies have reported that activation of the VEGF–VEGFR2 axis correlates with an immunosuppressive microenvironment. VEGF-VEGFR2 activation can induce the accumulation of immature dendritic cells, myeloid-derived suppressor cells, and regulatory T cells (Treg cells) and can inhibit the migration of T lymphocytes; these effects can be reversed by anti-angiogenic reagents [16–20]. Hence, at present, several clinical trials are underway to examine the effects of combined immunotherapy and anti-angiogenic treatments in various cancer types [18, 21]. However, the impact of anti-angiogenic reagents on the tumor immune microenvironment in human clinical samples remains unclear. Thus, this study aimed at using immunohistochemistry (IHC) and flow cytometry to investigate pre- and post-treatment tumor tissues obtained from patients with advanced GC receiving RAM-containing therapies.

## Methods

### Patients and samples

**Fig. 1:**
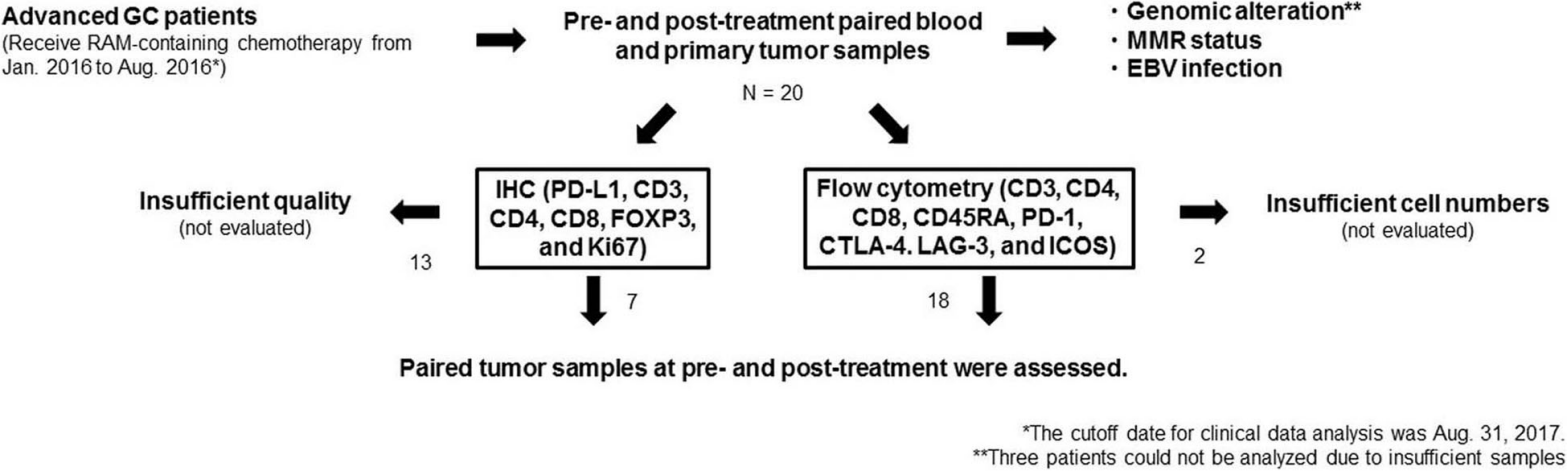
The scheme of our study. We prospectively enrolled patients with advanced GC who were scheduled to receive RAM-containing chemotherapy from January 2016 to August 2016. We obtained pre- and post-treatment paired blood and primary tumor samples by endoscopic biopsy to assess immune profiles from 20 patients. Archived FFPE samples were used for analyses of genomic alterations, MMR status, and EBV status. Tumor samples from 7 and 18 patients were subject to IHC and flow cytometry, respectively

In this study, we prospectively enrolled patients with advanced GC who were scheduled to receive RAM-containing chemotherapy between January 2016 and August 2016. In addition, we obtained pre- and post-treatment paired blood and primary tumor samples by endoscopic biopsy to assess immune profiles (Fig. 1). Peripheral blood mononuclear cells (PBMCs) were isolated by density gradient centrifugation with Ficoll-Paque (GE Healthcare, Little Chalfont, UK). Tumor tissues were minced and treated with gentleMACS Dissociator (Miltenyi Biotec, Bergisch Gladbach, Germany), as described previously [22], to collect tumor-infiltrating lymphocytes (TILs). This study was approved by the Institutional Review Board of the National Cancer Center and was conducted in accordance with international ethics guidelines, including the Declaration of Helsinki. We obtained written informed consent from all participants before sampling.

### Data collection

We reviewed medical records and collected patient data regarding clinicopathological features and treatment history. The cutoff date for analysis was August 31, 2017. All responses were assessed according to the RECIST v1.1 [23]. In addition, progression-free survival (PFS) was assessed as the time from treatment initiation to clinical or radiographic progression or death from any cause. Overall survival (OS) was defined as the period from treatment initiation to the time of death from any cause.

### Genomic analysis

DNA and RNA were extracted from archived FFPE tumor samples and were subjected to the Oncomine™ Comprehensive Assay version 3 (Thermo Fisher Scientific, Waltham, MA) which allowed to detect gene mutations, copy number variants and fusions across multiple genes (Additional file 1: Table S1). The detected genomic variant data were classified according to whether genetic drivers of cancer including gain- and loss-of-function mutations or single nucleotide variants based on the Oncomine Knowledgebase.

### Evaluation of mismatch repair (MMR) status

MMR status was examined with IHC with anti-mutLhomolog 1 (MLH1) mAb, anti-mutS homolog 2 (MSH2) mAb, anti-postmeiotic segregation increased 2 (PMS2) mAb, and anti-mutShomolog 6 (MSH6) mAb using archived FFPE tumor samples. Tumors were considered negative for MLH1, MSH2, PMS2, or MSH6 expression only if there was a complete absence of nuclear staining in the tumor cells, and normal epithelial cells and lymphocytes were employed as an internal control. Tumors lacking MLH1, MSH2, PMS2, or MSH6 expression were defined as MMR deficient, whereas tumors that maintained expression of all markers were considered MMR proficient.

### Evaluation of Epstein-Barr virus (EBV) infection

Chromogenic in situ hybridization (ISH) for EBV-encoded RNA was performed with fluorescein-labeled oligonucleotide probes (INFORM EBER probe) with enzymatic digestion (ISH protease 3, Ventana, Tucson, AZ) and an iViewBlue detection kit (Ventana) with use of the BenchMark ULTRA staining system. Archived FFPE tumor samples were used for the analysis.

### Immunohistochemistry for immune profiles

Biopsy specimens were formalin-fixed, paraffin-embedded, and sectioned onto slides for IHC, which was conducted using monoclonal antibodies against PD-L1 and CD8 using an automatic staining instrument (BenchMark ULTRA; Ventana). PD-L1 positivity was defined as staining in ≥1% in tumor cells or immune cells. CD8 staining intensity was assessed as follows: no staining (0), weak staining (1+), moderate staining (2+), and strong staining (3+). Two researchers (D. Kotani and T. Kuwata) independently evaluated the stained slides. Multiplexed fluorescent immunohistochemistry was performed by the TSA method using an Opal IHC kit (PerkinElmer, Waltham, MA) according to the manufacturer’s instructions. Multiplexed fluorescent labeled images of three randomly selected fields (669 × 500 micrometer) were captured with an automated imaging system (Vectra ver3.0, PerkinElmer).

### Flow cytometry analysis

We washed cells in PBS with 2% fetal calf serum and then stained with mAbs specific for CD3, CD4, CD8, CD45RA, PD-1, LAG-3, ICOS, VEGFR2, or CTLA-4 as well as a fixable viability dye. In addition, intracellular Ki67 and FOXP3 staining was performed with an anti-FOXP3 mAb and FOXP3 Staining Buffer Set (eBioscience) according to the manufacturer’s protocol. After washing, we analyzed all cells with the LSRFortessa instrument (BD Biosciences) and FlowJo software (TreeStar, Ashland, OR). Antibody dilutions were performed per the manufacturer’s instructions.

### Antibodies and reagents

Alexa Fluor 700–conjugated anti-CD3 (UCTH1) mAb, violet 500-conjugated anti-CD4 (RPA-T4) mAb, and Brilliant Violet 421 (BV421)–conjugated anti-PD-1 (EH12) mAb were obtained from BD Biosciences (Franklin Lakes, NJ). BV785-conjugated anti-CD8 (RPA-T8) mAb, BV711-conjugated anti-CD45RA (HI100) mAb, and APC-conjugated anti-CTLA-4 (L3D10) mAb were purchased from BioLegend (San Diego, CA). Phycoerythrin (PE)-conjugated anti-FOXP3 (Forkhead Box P3, 236A/E7) mAb, PE- and Cy7-conjugated anti-ICOS (3D12) mAbs, peridinin–chlorophyll–protein complex (PerCP)- and Cy5.5-conjugated anti-Ki67 (20Raj1) mAb, and eFluor 780-conjugated fixable viability dye were obtained from eBioscience (Santa Clara, CA). Fluorescein isothiocyanate (FITC)–conjugated anti-LAG-3 (17B4) mAb was obtained from ENZO Life Science (Farmingdale, NY). Anti-CD4 (4B12) mAb, anti-Ki67 (MIB-1) mAb, anti-MLH1 (ES05) mAb, anti-MSH2 (FE11) mAb, anti-PMS2 (EP51) mAb, and anti-MSH6 (EP49) mAb for IHC were obtained from Dako (Copenhagen, Denmark). Anti-PD-L1 (SP263) mAb and anti-CD8 (SP57) mAb for IHC were obtained from Ventana. Anti-CD3 (SP7) mAb and anti-FOXP3 (236A/E7) mAb for IHC was purchased from Abcam; RAM was obtained from Eli Lilly and Company (Indianapolis, IN); and VEGFA was obtained from Wako (Osaka, Japan). In addition, APC was conjugated to RAM to detect VEGFR2.

### Statistical analysis

We used Welch’s or paired *t*-tests to analyze continuous variables. All survival curves were estimated using the Kaplan–Meier method and compared using the log-rank test. All statistical analyses were two-tailed and were performed using Prism version 7 software (GraphPad Software, Inc., La Jolla, CA). *P* < 0.05 was considered statistically significant.

## Results

### Patient characteristics

A total of 20 patients (age, 46–79 years; median, 67 years; men, 13; women, 7) with advanced GC and with available pre- and post-treatment samples that were scheduled to receive RAM-containing chemotherapy were enrolled in this study (Fig. 1). All clinical characteristics are summarized in Additional file 2: Table S2 and Additional file 3: Table S3. In all, 11 patients had an intestinal subtype, and 9 patients had a diffuse histology; all exhibited a good performance status (PS, 0 or 1). A majority of patients (14/20) received RAM-containing therapy as a second-line therapy. Individual treatment regimens also comprised paclitaxel (10/20), nab-paclitaxel (2/20), irinotecan (5/20), or RAM monotherapy (3/20). A total of 6 patients (30%) attained a partial response (PR), 11 (55%) had stable disease (SD), and 3 (15%) had progressive disease (PD). The median PFS and OS were 110 and 324 days, respectively. These clinical data were consistent with those of a previous study [13]. Typically, patients were administered 8 mg/kg RAM intravenously on days 1 and 15 of a 28-day cycle, and imaging was performed every 6–8 weeks to assess antitumor response. Pre-treatment biopsies were conducted within 2 weeks of initiation of RAM-containing therapies. Post-treatment biopsies were performed along with each imaging assessment if possible. The median number of times the biopsy was performed in each patient was 3 (range, 2–6).

### Genomic alterations, MMR status, and EBV infection

A recent study has shown that ICB exhibits efficacy against patients with MMR deficient or EBV-positive GC from comprehensive genomic analyses [24]. We then analyzed genomic alterations, MMR status and EBV infection and all data are summarized in Additional file 4: Figure S1 and Additional file 3: Table S3 although 3 patients could not be assessed for NGS due to insufficient samples. *TP53* were frequently mutated (10/17) and *ERBB2*, *MET*, *FGFR2*, or *KRAS*, amplification, or a *RHOA* mutation were also identified, which was in line with a previous study [25]. In contrast, all were MMR proficient GC and only one was EBV-positive GC (Additional file 2: Table S2 and Additional file 3: Table S3).

### PD-L1 expression and CD8^+^ T-cell infiltration after RAM-containing therapies

**Fig. 2:**
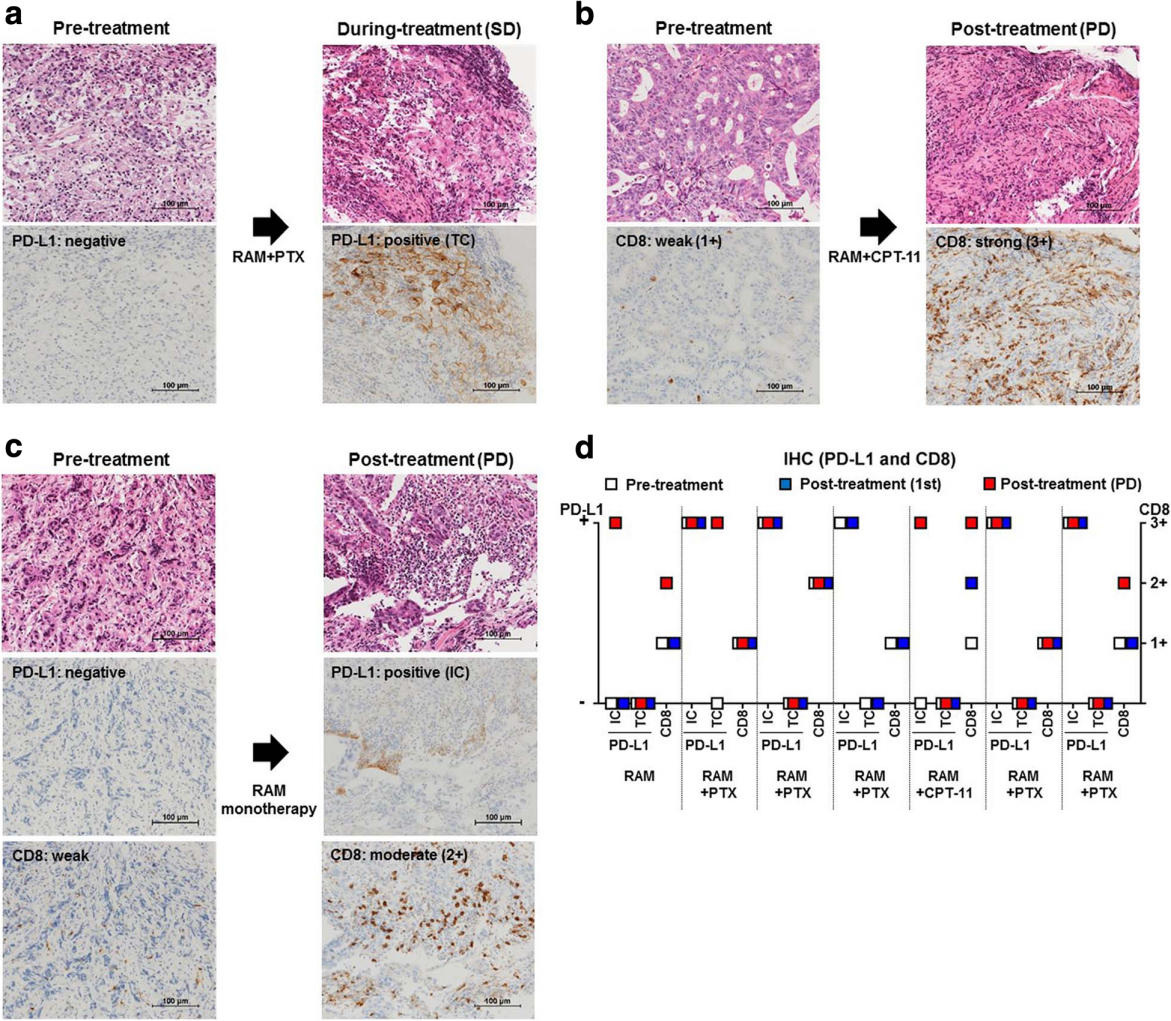
PD-L1 expression and CD8^+^ T-cell infiltration by IHC. **a** Hematoxylin–eosin (HE) staining and IHC for PD-L1 in a 67-year-old male with advanced GC. The pre-treatment sample was negative for PD-L1 in both tumor cells and immune cells. After 4 weeks of RAM and PTX treatment, the patient attained SD, and tumor cells were positive for PD-L1. **b** HE staining and IHC for CD8 in a 79-year-old female with advanced GC. At pre-treatment, CD8 staining was weak (1+). After 10 weeks of RAM and CPT-11 treatment, the patient experienced PD, and CD8 staining was strong (3+). **c** HE staining and IHC for PD-L1 and CD8 in a 73-year-old female with advanced GC. The pre-treatment sample was negative for PD-L1 in both tumor cells and immune cells, while CD8 staining was weak (1+). After 23 weeks of RAM monotherapy, the patient experienced PD. Immune cells in the post-treatment sample were PD-L1-positive; CD8 staining was moderate (2+). **d** Summary of IHC. The PD-L1 status in tumor and immune cells changed to positive in 3 of 7 patients post-treatment [1, tumor cells positive (1/7); 2, immune cells positive (7/7). CD8^+^ T-cell infiltration was weak (1+) in 6 patients and moderate (2+) in 1 patient prior to the initiation of therapy. CD8^+^ T-cell infiltration was increased in 3 patients. Both PD-L1 expression and CD8^+^ T-cell infiltration increased after treatment in 2 patients. TC, tumor cells; IC. immune cells

We next used IHC to evaluate PD-L1 expression and CD8^+^ T-cell infiltration in tumor samples. Paired tumor samples (*n* = 7) with sufficient quality at pre-treatment, first evaluation, and PD were assessed for changes induced by RAM-containing therapies (Fig. 1). Prior to the initiation of therapy, none of the tumor cells were positive for PD-L1, while immune cells from 5 of 7 patients expressed PD-L1. The PD-L1 status in tumor and immune cells changed to positive in 3 of 7 patients post-treatment [1, tumor cells positive (1/7); 2, immune cells positive (7/7); Fig. 2a, c, and d]. CD8^+^ T-cell infiltration was weak (1+) in 6 patients and moderate (2+) in 1 patient prior to the initiation of therapy. An incremental increase in CD8^+^ T-cell infiltration was observed in 3 patients (Fig. 2b, c, and d). Both PD-L1 expression and CD8^+^ T-cell infiltration increased after treatment in 2 patients, suggesting that PD-L1 elevation may be due to IFN-γ exposure during CD8^+^ T-cell attack [26] (Fig. 2c and d). PD-L1 expression and CD8^+^ T-cell infiltration were enhanced with any RAM-containing therapies regardless of additional cytotoxic reagents such as paclitaxel (PTX) and irinotecan (CPT-11) (Fig. 2d and Additional file 3: Table S3).

### Accumulation of effector Treg cells in TILs

**Fig. 3:**
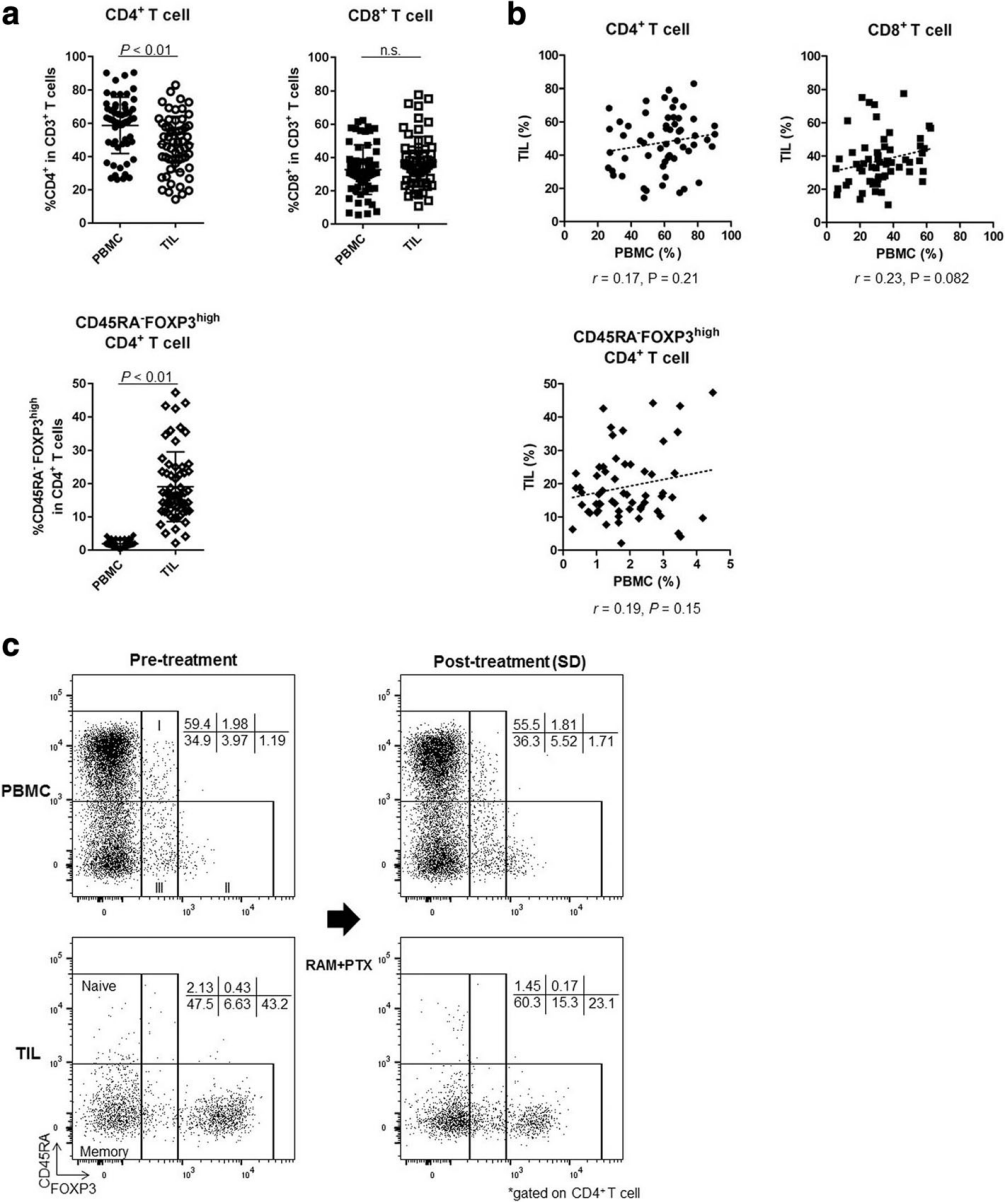
Difference of T-cell subsets between PBMCs and TILs. **a** Comparison of T-cell subsets between PBMCs and TILs (total of 59 samples from 18 patients). The frequency of CD4^+^ T cells was marginally higher in PBMCs than in TILs (58.77 ± 16.94% vs. 47.59 ± 16.68%; *P* < 0.01). The CD8^+^ T-cell frequency was similar in PBMCs and TILs (32.70 ± 14.89% vs. 37.39% ± 15.28%; *P* = 0.059). The frequency of eTreg cells was very low in PBMCs but was significantly higher in TILs (1.89 ± 1.0 vs. 19.05 ± 10.48; *P* < 0.01). n.s., not significant. **b** Correlation of T-cell subsets in PBMCs and TILs (total of 59 samples). The frequencies of CD4^+^ T cells, CD8^+^ T cells, and eTreg cells in TILs did not correspond with those in PBMCs (*r* = 0.17, 0.23, and 0.19; *P* = 0.21, 0.082, and 0.15, respectively). **c** Representative flow cytometry plots of PBMCs and TILs both pre- and post-treatment in the same patient. This patient was treated with RAM and PTX and attained SD after 5 weeks of treatment. eTreg cells were particularly enriched in TILs compared with PBMCs. Furthermore, TIL eTreg cells decreased following treatment (from 43.2 to 23.1%), while PBMC eTreg cells did not change significantly (from 1.19 to 1.71%)

The upregulation of FOXP3 upon TCR stimulation of conventional T cells compromises the precise identification of CD4^+^ Treg cells in humans [27]. Thus, we proposed a classification of human Treg cells based on expression levels of both FOXP3 and the naïve marker CD45RA [28, 29]. Hence, FOXP3^+^CD4^+^ T cells can be divided into 3 categories: I, naïve Treg cells (CD45RA^+^FOXP3^low^CD4^+^); II, effector Treg cells (eTreg cells; CD45RA^−^FOXP3^high^CD4^+^); and III, non-Treg cells (CD45RA^−^FOXP3^low^CD4^+^; Fig. 3). Notably, eTreg cells display a highly suppressive phenotype. Owing to difficulties in the precise identification of this fraction by IHC, we employed flow cytometry to analyze TIL immune profiles in detail, with particular emphasis on Treg cells [22]. We could not evaluate samples from 2 patients because of a lack of sufficient cell numbers (Fig. 1). Thus, 59 samples of both PBMCs and TILs from 18 patients were analyzed. The CD4^+^ T-cell frequency was marginally higher in PBMCs than in TILs (58.77% ± 16.94% vs. 47.59% ± 16.68%; *P* < 0.01), while the CD8^+^ T-cell frequency was comparable in both PBMCs and TILs (32.70% ± 14.89% vs. 37.39 ± 15.28%; *P* = 0.059; Fig. 3a). The frequency of eTreg cells was significantly higher in TILs compared with PBMCs (1.89 ± 1.0 vs. 19.05 ± 10.48; *P* < 0.01; Fig. 3a and c). Furthermore, the frequencies of CD4^+^ T cells, CD8^+^ T cells, and eTreg cells in TILs were not reflected in PBMCs (*r* = 0.17, 0.23, and 0.19; *P* = 0.21, 0.082, and 0.15, respectively; Fig. 3b). These findings suggest that analyses of TILs, where T cells directly attack tumor cells, are more important than analyses of PBMCs in investigating the principles of cancer immunology.

### Reduction of eTreg cells in TILs after RAM-containing therapies

**Fig. 4:**
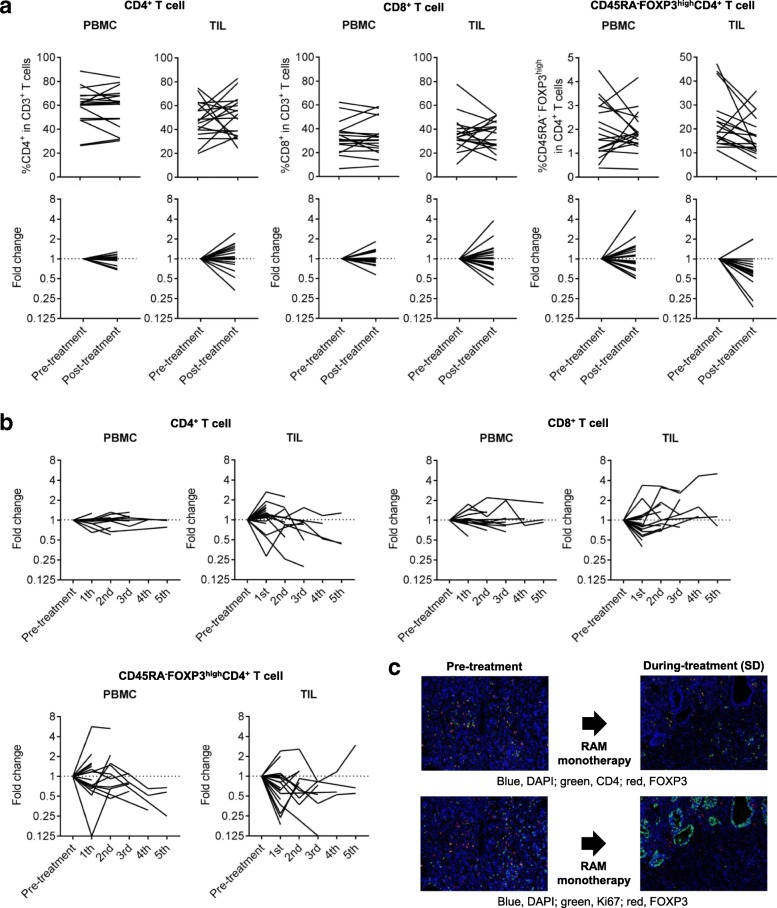
Reduction in TIL eTreg cells after RAM-containing therapies. **a** Kinetic changes of CD4^+^ T cells, CD8^+^ T cells, and eTreg cells. The frequency of eTreg cells in TILs significantly decreased following RAM-containing therapies (22.67 ± 11.19% vs. 16.33 ± 8.44%; *P* = 0.034), while no significant difference was observed in eTreg cells in PBMCs (1.97 ± 1.15% vs. 1.89 ± 0.85%; *P* = 0.74). There was no significant difference in the frequency of CD4^+^ T cells and CD8^+^ T cells between pre- and post-treatment. The averages were used if more than one samples at post-treatment were available. n.s., not significant. **b** % of changes from base line of CD4^+^ T cells, CD8^+^ T cells, and eTreg cells. The kinetic changes are more dynamic in TILs compared with PBMCs especially in CD4^+^ T cells and CD8^+^ T cells. eTreg cells showed a trend to decrease in TIL. **c** IHC for CD4, FOXP3 and Ki67. FOXP3^+^CD4^+^ T cells and Ki67^+^FOXP3^+^CD4^+^ T cells were reduced after RAM monotherapy. Blue, DAPI; green, CD4 (upper) or Ki67 (lower); red, FOXP3

We focused on eTreg cells in TILs because Treg cells, which reportedly correlate with poor prognosis in many cancers [22, 29, 30], were significantly accumulated in TILs. We did not observe any correlations between eTreg frequency and patient characteristics, including age, sex, histology, and line of treatment (Additional file 5: Figure S2). In addition, no correlation between eTreg frequency and *TP53* mutations or receptor tyrosine kinase/MAPK/PI3K -related gene alterations was observed (Additional file 5: Figure S2). Figure 4a and b summarizes the kinetic changes in CD4^+^ T-cells, CD8^+^ T-cells, and eTreg cells across all patients, demonstrating that the kinetic changes are more dynamic in TILs compared to PBMCs especially in CD4^+^ T cells and CD8^+^ T cells. In cases where patients received multiple post-treatment biopsies, we calculated the average of all post-treatment samples. The frequency of eTreg cells was significantly decreased in TILs after treatment (pre-treatment: 22.67% ± 11.19% vs. post-treatment: 16.33% ± 8.44%; *P* = 0.034); however, no significant difference was observed in eTreg cells in PBMCs (1.97% ± 1.15% vs. 1.89% ± 0.85%; *P* = 0.74; Fig. 4a). This trend was observed in both patients receiving RAM monotherapy and RAM with chemotherapy regardless of additional cytotoxic reagents (Additional file 6: Figure S3). The frequency of eTreg cells in CD3^+^ TILs tended to decrease after the treatment (pre-treatment: 11.41% ± 8.57% vs. post-treatment: 8.34% ± 5.78%; *P* = 0.19) while that of CD45RA^−^FOXP3^−^CD4^+^ T cells in CD3^+^ TILs was comparable during RAM-containing therapy (pre-treatment: 28.75% ± 9.94% vs. post-treatment: 28.65% ± 7.21%; *P* = 0.97), (Additional file 7: Figure S4). To further confirm the reduction of Treg cells, multi-color IHC was performed, showing that FOXP3^+^CD4^+^ T cells with the reduction of proliferating Treg cells detected by Ki67^+^FOXP3^+^CD4^+^ T cells clearly decreased (Fig. 4c).

### Reduction of PD-1 expression by CD8^+^ T cells in TILs after RAM-containing therapies

**Fig. 5:**
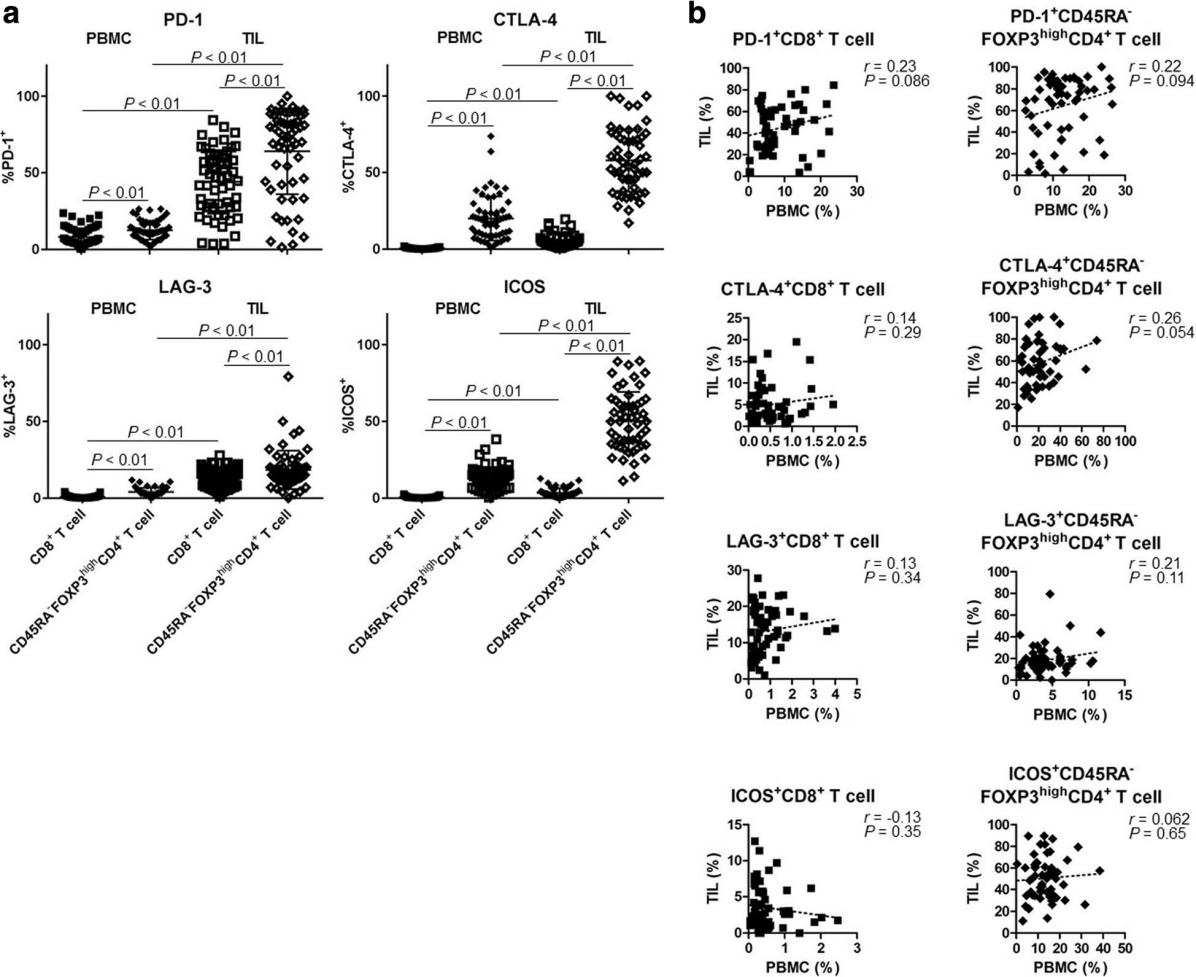
High expression of IC molecules in TILs. **a** A comparison of the expression levels of IC molecules in peripheral blood and tumor tissue (total of 59 samples from 18 patients). IC molecules (PD-1, CTLA-4, LAG-3, and ICOS) were highly expressed in TILs compared with PBMCs. All of them were higher in CD45RA^−^FOXP3^high^CD4^+^ T cells (eTreg cells) than in CD8^+^ T cells (PD-1^+^CD8^+^ T cells and PD-1^+^ eTreg cells in PBMCs and in TILs [8.12% ± 5.75% vs. 12.5% ± 6.17% vs. 44.18% ± 20.7% vs. 64.01% ± 28.02%, respectively, *P* < 0.01 across all categories]; CTLA-4^+^CD8^+^ T cells and CTLA-4^+^ eTreg cells in PBMCs and in TILs [0.52% ± 0.45% vs. 20.0% ± 14.35% vs. 5.01% ± 4.35% vs. 57.96% ± 20.54%, respectively, *P* < 0.01 across all categories]; LAG-3^+^CD8^+^ T cells and LAG-3^+^ eTreg cells in PBMCs and in TILs [0.79% ± 0.78% vs. 4.05% ± 2.54% vs. 13.14% ± 6.31% vs. 18.33% ± 12.61%, respectively, *P* < 0.01 across all categories]; ICOS^+^CD8^+^ T cells and ICOS^+^ eTreg cells in PBMCs and in TILs [0.54% ± 0.51% vs. 12.94% ± 7.15% vs. 3.47% ± 2.89% vs. 50.31% ± 19.09%, respectively, *P* < 0.01 across all categories]). **b** Correlation of the expression of IC molecules in PBMCs and TILs (total of 59 samples from 18 patients). No correlation was observed between PBMCs and TILs with regard to the expression of IC molecules by CD8^+^ T cells or eTreg cells (PD-1, CTLA-4, LAG-3, and ICOS in CD8^+^ T cells, *r* = 0.23, 0.14, 0.13, and − 0.13, *P* = 0.086, 0.29, 0.34, and 0.35, respectively; in eTreg cells, *r* = 0.22, 0.26, 0.21, and 0.062, *P* = 0.094, 0.054, 0.11, and 0.65, respectively)

In addition to eTreg cells, we assessed the expression profiles of IC molecules (PD-1, CTLA-4, LAG-3, and ICOS). IC molecules were expressed at significantly higher levels by CD8^+^ T cells and eTreg cells in TILs compared with those in PBMCs (Fig. 5a). Notably, eTreg cells exhibited higher expression of all IC molecules compared with CD8^+^ T cells (Fig. 5a). In particular, ICOS was expressed by eTreg cells in TILs in accordance with our previous study (Fig. 5a) [30]. Furthermore, we observed no correlation between PBMCs and TILs with regard to the expression of these IC molecules by CD8^+^ T cells or eTreg cells (PD-1, CTLA-4, LAG-3, and ICOS in CD8^+^ T cells: *r* = 0.23, 0.14, 0.13, and − 0.13, *P* = 0.086, 0.29, 0.34, and 0.35, respectively; eTreg cells: *r* = 0.22, 0.26, 0.21, and 0.062, *P* = 0.094, 0.054, 0.11, and 0.65, respectively; Fig. 5b). These results suggest that IC molecules on Treg cells in TILs could be potential therapeutic targets and that ICB may affect Treg cells.

**Fig. 6:**
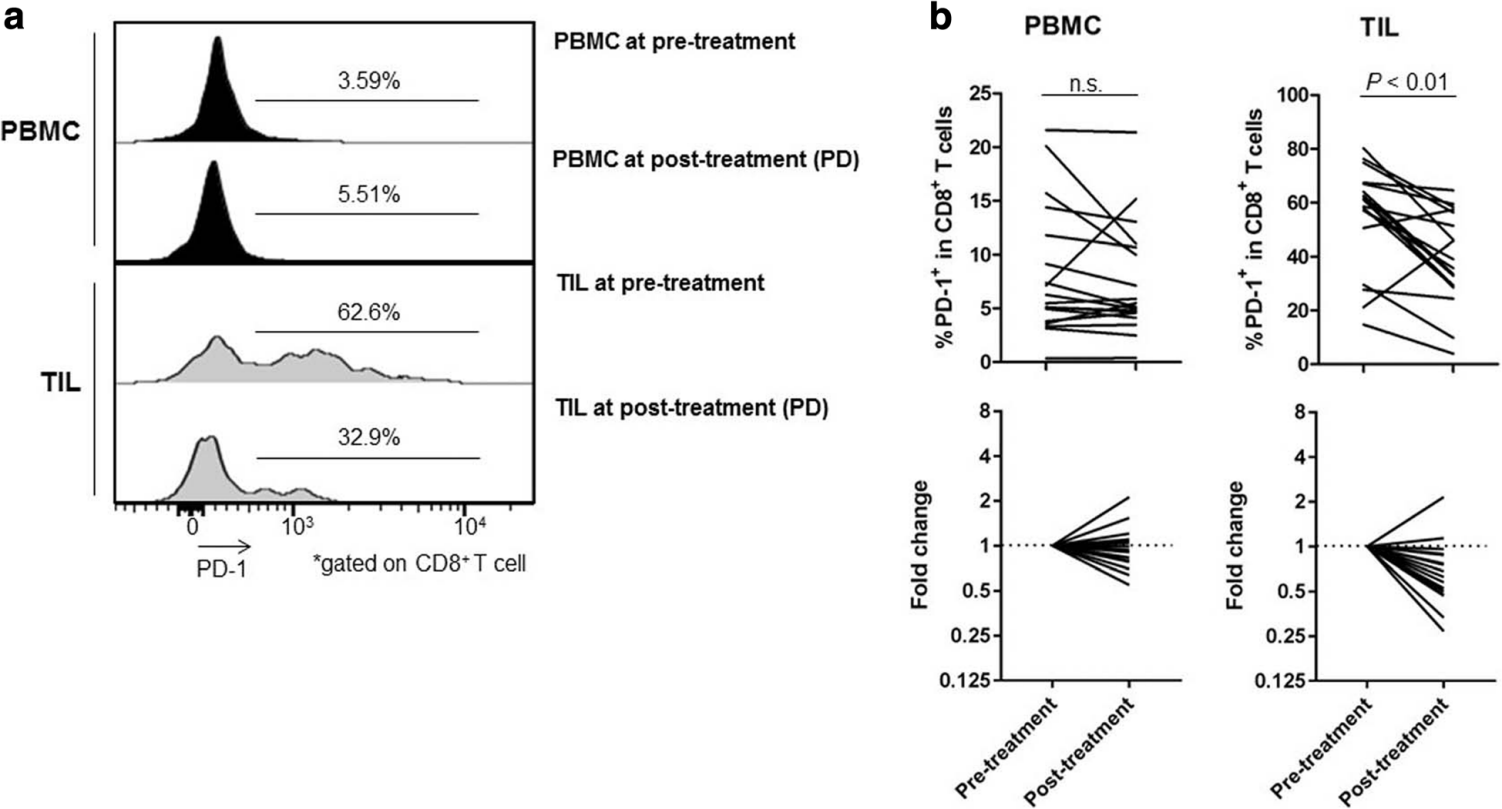
Reduction of PD-1 expression by CD8^+^ T cells after RAM-containing therapies. **a** Representative flow cytometry from PBMCs and TILs both pre- and post-treatment in the same patient. This patient was treated with RAM and CPT-11 and attained PD after 6 weeks of treatment. The PD-1 expression by CD8^+^ T cells in TILs was especially elevated as compared to that in PBMCs. PD-1 expression by CD8^+^ T cells were decreased after treatment (from 62.6 to 32.9%) in TILs, while these cells were not decreased in PBMCs (from 3.59 to 5.51%). **b** Kinetic changes in PD-1 expression by CD8^+^ T cells. The PD-1 expression by CD8^+^ T cells in TILs was significantly decreased after RAM-containing therapies (54.99 ± 19.06% vs. 39.23 ± 17.15%; *P* < 0.01), whereas no significant difference was observed in PD-1 expression by CD8^+^ T cells in PBMCs (8.25 ± 6.05% vs. 7.47 ± 5.18%; *P* = 0.33). The averages were used if more than one samples at post-treatment were available. n.s., not significant

The expression of IC molecules in several T-cell subpopulations exhibited dynamic changes (Additional file 8: Figure S5, Additional file 9: Figure S6, Additional file 10: Figure S7). Among these IC molecules, PD-1 expression by CD8^+^ T cells was significantly reduced in TILs after RAM-containing therapies (54.99% ± 19.06% vs. 39.23% ± 17.15%; *P* < 0.01; Fig. 6a and b) in both patients receiving RAM monotherapy and RAM with chemotherapy regardless of additional cytotoxic reagents (Additional file 6: Figure S3). In contrast, no significant difference was observed between pre- and post-treatment PBMCs (8.25% ± 6.05% vs. 7.47% ± 5.18%; *P* = 0.33; Fig. 6a and b). These results suggest that PD-1 expression by CD8^+^ T cells are reduced by RAM-containing therapies, especially in TILs. In contrast, ICOS was highly expressed by eTreg cells in TILs, but there was no significant difference between pre- and post-treatment (PBMCs: 12.77% ± 5.73% vs. 13.07% ± 5.80%; *P* = 0.83 and TILs: 50.71% ± 14.42% vs. 53.76% ± 17.05%; *P* = 0.45; Additional file 11: Figure S8).

### Prognostic significance of eTreg frequency in patients receiving RAM-containing therapies

**Fig. 7:**
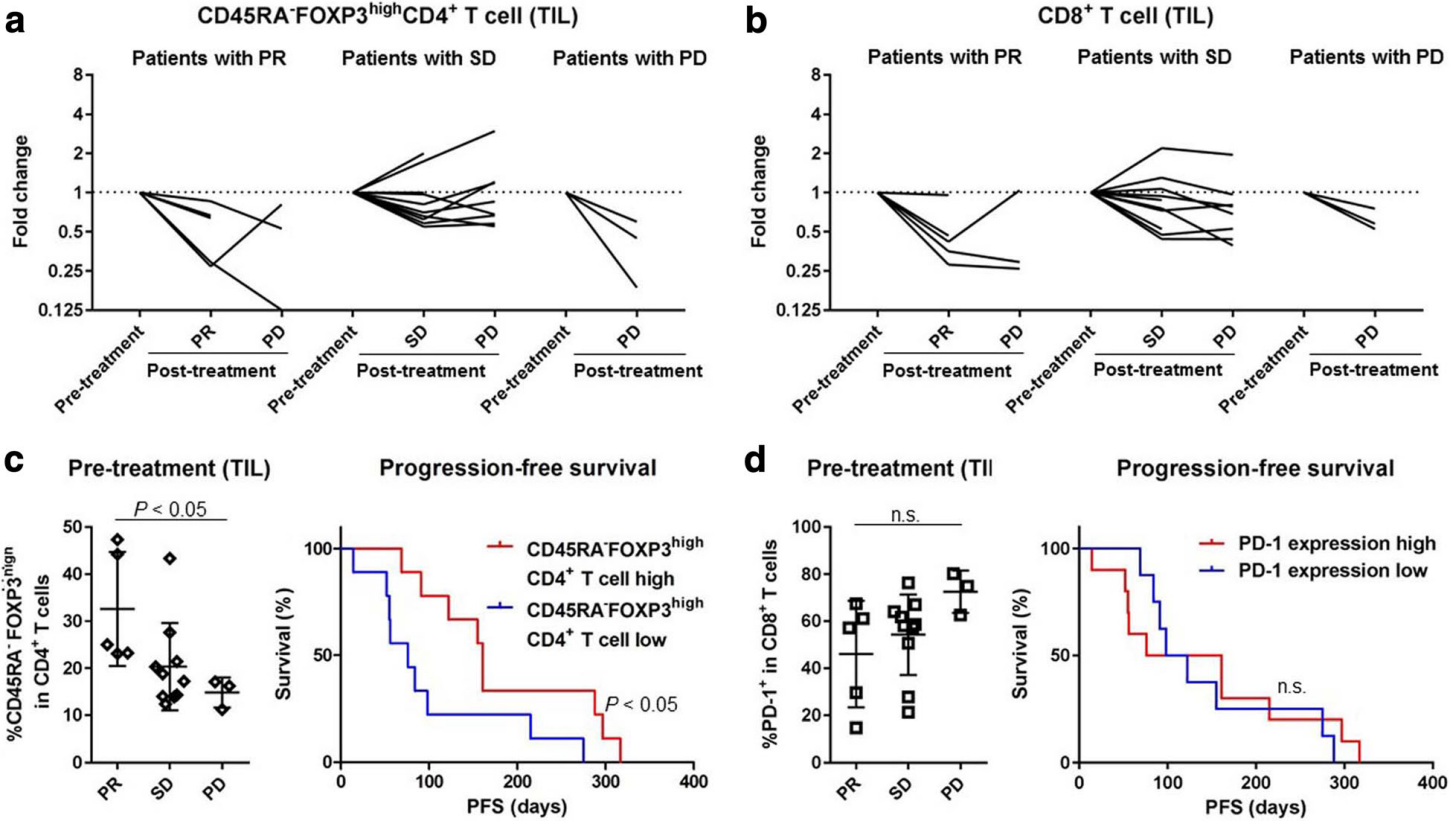
Prognostic significance of CD45RA^−^FOXP3^high^CD4^+^ T cells (eTreg cells) in TILs. **a** Kinetic changes (% of change from the base line) in eTreg cells according to clinical response. In all 5 PR patients, eTreg cells in TILs were decreased at the PR stage compared with pre-treatment, whereas no trend was observed between the PR and PD stages (1 increased from PR to PD; 2 decreased from PR to PD). In 9 SD patients, no trend was observed in terms of eTreg-cell kinetic changes. In all 3 patients who experienced PD at the first evaluation, eTreg cells in TILs were decreased at the PD stage compared with the pre-treatment time point. The averages were used if more than one samples at post-treatment were available. **b** Kinetic changes (% of change from the base line) in PD-1 expression by CD8^+^ T cells according to clinical responses. In almost all patients, PD-1 expression by CD8^+^ T cells in TILs were decreased following treatment; this did not correlate with the clinical response. **c** eTreg cells and clinical outcomes. Patients with PR had a significantly higher frequency of eTreg cells in TILs than those with PD (32.56 ± 12.11% vs. 14.83 ± 3.18%; *P* = 0.036), whereas no significant difference was observed between patients with PR and SD or SD and PD (32.56% ± 12.11% vs. 20.32% ± 9.28%, *P* = 0.094 and 20.32% ± 9.28% vs. 14.83% ± 3.18%, *P* = 0.14). Patients with a high frequency of eTreg cells in pre-treatment TILs had a significantly longer PFS than those with a low frequency (161 days vs. 76 days; hazard ratio, 0.30; *P* = 0.032). **d** PD-1 expression by CD8^+^ T cells and clinical responses. Both patients with PR and patients with SD tended to have a lower PD-1 expression by CD8^+^ T cells in TILs than those with PD (46.00% ± 22.65% and 54.23% ± 17.11% vs. 72.50% ± 8.97%, *P* = 0.067 and 0.051, respectively), but the difference was not significant. No significant difference was observed in the PFS between patients with a high PD-1 expression by CD8^+^ T cells in pre-treatment TILs and those with a low expression (118.5 days vs. 110 days; hazard ratio, 0.83; *P* = 0.72). n.s., not significant

As RAM-containing therapies decreased both eTreg cells and PD-1 expression by CD8^+^ T cells in TILs, we assessed whether the frequencies of these cells corresponded with clinical responses and thus whether either frequency could be a biomarker for stratifying clinical responders. In our cohort, 5 patients attained PR, 3 of whom received biopsies at both the PR and PD stages. TIL eTreg cells were decreased at the PR stage compared with pre-treatment in all 5 patients, (Fig. 7a), though there was no correlation with clinical responses (1 increased at PD from PR; 2 decreased at PD from PR). In patients with SD (*n* = 7), we observed no trends in the eTreg kinetic changes (Fig. 7a). In all 3 patients who experienced PD at first evaluation, TIL eTreg cells were reduced at the PD stage compared with pre-treatment (Fig. 7a). Likewise, PD-1 expression by CD8^+^ T cells in TILs were more likely to be decreased post-treatment, which did not correlate with clinical responses (Fig. 7b). These findings suggest that the reduction in TIL eTreg and PD-1 expression by CD8^+^ T cells caused by RAM-containing therapies is independent of clinical outcomes.

We then assessed the prognostic significance of eTreg cells and PD-1 expression by CD8^+^ T cells in pre-treatment TILs. Patients with PR had a significantly higher frequency of eTreg cells in TILs than those with PD (32.56% ± 12.11% vs. 14.83% ± 3.18%; *P* = 0.036; Fig. 7c). There was no significant difference in % of eTreg-cell reduction among patients with PR, SD, and PD (15.41% ± 12.31% vs. 1.21% ± 10.7% vs. 8.37% ± 1.61%; *P* = 0.063 and 0.27, respectively; Additional file 12: Figure S9). Further, patients with a high frequency of eTreg cells in pre-treatment TILs had a significantly longer PFS than those with a low frequency of eTreg cells (161 days vs. 76 days; *P* = 0.032; Fig. 7c). Conversely, no significant difference was observed in the OS of these two groups (324 days vs. 350 days; *P* = 0.71). Although not significant, patients with PR tended to have a lower PD-1 expression by CD8^+^ T cells in pre-treatment TILs than those with PD (46.0% ± 22.65% vs. 72.5% ± 8.97%; *P* = 0.067; Fig. 7d), but there was no significant difference in % of PD-1 reduction by CD8^+^ T cells among patients with PR, SD, and PD (17.43% ± 11.23% vs. 11.66% ± 18.28% vs. 27.37% ± 7.96; *P* = 0.46 and 0.20, respectively; Additional file 12: Figure S9), and no significant difference was observed in the PFS between patients with a high and low pre-treatment PD-1 expression by CD8^+^ T cells in TILs (118.5 days vs. 110 days; *P* = 0.72; Fig. 7d). These results suggest that patients with a high frequency of eTreg cells in TILs, which can be decreased by RAM, exhibit a favorable response to RAM-containing therapies.

### Impact of anti-VEGFR2 blockade on eTreg cells in vitro

Our clinical data revealed that RAM significantly reduces Treg cells in the tumor microenvironment. To gain insight into the cellular mechanism of eTreg-cell reduction, we assessed VEGFR2 expression in various types of T cells. VEGFR2 was highly expressed by eTreg cells compared with other fractions, as previously reported (Additional file 13: Figure S10) [31]. In addition, VEGFR2^+^ eTreg cells had higher Ki67 expression compared with VEGFR2^−^ eTreg cells (Additional file 13: Figure S10), suggesting a significant proliferative capacity in VEGFR2^+^ eTreg cells. When PBMCs were treated with VEGFA, a VEGFR2 ligand, eTreg cells (and in particular VEFR2^+^ eTreg cells) proliferated (Additional file 13: Figure S10). Further, the eTreg cells that were increased by VEGFA could be decreased by RAM treatment (Additional file 13: Figure S10). In contrast to eTreg cells, VEGFR2^+^CD8^+^ T cells exhibited slightly higher PD-1 expression than VEGFR2^−^CD8^+^ T cells, and PD-1 expression by CD8^+^ T cells did not increase after VEGFA treatment (Additional file 13: Figure S10). We propose that VEGFR2 is highly expressed by eTreg cells and that VEGFA stimulates VEGFR2^+^ eTreg cell proliferation, which can be overcome by RAM.

## Discussion

Recent studies have demonstrated that cancer immunotherapies, particularly those that modulate immune co-inhibitory signals, can be beneficial for patients with various types of cancers [1–7]. However, limited clinical efficacies necessitate the development of better therapies, including the identification of biomarker(s) and the formulation of combinatorial therapies with improved clinical efficacies. Tumor PD-L1 expression correlates with favorable clinical responses to anti-PD-1/PD-L1 antibodies [6, 32]; however, CD8^+^ T-cell infiltration is also critical for tumor immune responses [33, 34]. Intratumoral PD-L1 expression can be induced either by constitutive oncogenic signaling pathways or chromosomal alterations (innate resistance) or through IFN-γ signaling stimulated by host defense mechanisms, such as CD8^+^ T cells in tumors (acquired resistance). This indicates a probable correlation between CD8^+^ T cells and PD-L1 expression. VEGF is related to the immunosuppressive microenvironment in that it inhibits T-cell proliferation and infiltration; this inhibition can be reversed by anti-angiogenic reagents, including RAM [35, 36]. Both CD8^+^ T-cell infiltration and PD-L1 expression were induced by RAM-containing therapies. In addition, TIL eTreg cells and PD-1 expression by CD8^+^ T cells are decreased by RAM-containing therapies. To the best of our knowledge, this is the first study to demonstrate that an anti-angiogenic reagent can cause an increase in CD8^+^ T-cell infiltration and PD-L1 expression and a decrease in eTreg cells and PD-1 expression by CD8^+^ T cells in the tumor microenvironment in humans. PD-1 expression by TIL CD8^+^ T cells was decreased by RAM-containing therapies, which is in line with findings in an animal model demonstrating the effects of VEGFA/VEGFR2 on the expression of IC molecules [17]. Furthermore, this study also demonstrated that the pre-treatment TIL eTreg frequency could be a biomarker for stratifying clinical responders to RAM-containing therapies. Corroborating our results, a previous pre-clinical animal study reported the efficacy of combining anti-PD-1/PD-L1 blocking therapies and anti-angiogenic reagents [17]. Thus, this strategy is a promising combination, and several clinical trials have been pursued with favorable outcomes [20].

While systemic chemotherapy is the primary treatment option for patients with unresectable advanced GC, which is the fifth leading cancer worldwide [37], their prognosis remains poor, with a median survival time of approximately 1 year in those receiving conventional therapy [38–40]. The combination of trastuzumab, an antibody targeting human epidermal growth factor receptor type 2 (HER2), and chemotherapy has reportedly increased survival in patients with HER2-positive gastric or gastro-esophageal junction cancer [38], although HER2-positive tumors only account for 7–17% of GCs [41–43]. Based on the findings of two pivotal global phase III trials, REGARD and RAINBOW, RAM has become one of the standard chemotherapies for GC, though the absolute improvements provided by this therapy remain limited [12, 13]. A recent phase III trial demonstrated a survival benefit in advanced GC patients receiving nivolumab after two or more previous lines of chemotherapy. This resulted in the approval of nivolumab for the treatment of GC in Japan [7], although its therapeutic efficacy remains unsatisfactory. Another study has shown that ICB exhibits efficacy against patients with MMR deficient or EBV-positive GC from comprehensive genomic analyses [24]. In our cohort, however, only one patient had EBV-positive GC and no one had MMR deficient GC. Thus, the relationship between these factors and immune status warrants to study with a large patient cohort. In contrast, our study suggests that a combination strategy using anti-angiogenic reagents and ICB may be a promising therapeutic approach for GC. Furthermore, several studies have already demonstrated the efficacy of both anti-angiogenic reagents and ICB in several other types of cancer, including NSCLC, renal cell carcinoma, and bladder cancer [5, 6, 8, 9, 14, 15]. Moreover, similar combination strategies have already been tested in clinical trials targeting these cancer types [18, 21].

We employed flow cytometry to investigate the detailed immune profiles of hosts, where significant differences exist between PBMCs and TILs. The RAM-containing therapy-induced reduction in TIL eTreg cells and PD-1 expression by CD8^+^ T cells suggests the importance of analyzing TILs, in which T cells directly attack tumor cells. The decrease of eTreg cells and PD-1 expression by CD8^+^ T cells in TILs in some PR patients may be a reflection of reduced tumor burdens, whereas this tendency was also observed at PD state, suggesting that RAM-containing therapies can reduce eTreg cells and PD-1 expression by CD8^+^ T cells in TILs independently of clinical responses. eTreg cells are a highly immunosuppressive subset of CD4^+^ T cells characterized by high expression of the master regulatory transcription factor FOXP3 [44–46]. Our study demonstrates that a high eTreg-cell frequency in TILs correlates with a better response to RAM-containing therapies. Thus, TIL eTreg cell frequency could be a biomarker for stratifying clinical responders. Considering the role of Treg cells in promoting tumor progression by suppressing antitumor immunity, the RAM-induced reduction in Treg cells could be more important for clinical responses to RAM-containing therapies in patients with a high eTreg frequency (in whom tumor progression should be highly dependent on eTreg cells) than in patients with a low eTreg frequency. Indeed, patients with PR, although not significant, tended to have higher eTreg-cell reduction.

Myeloid cells including myeloid-derived suppressor cells in tumors has been implicated in suppression of antitumor immunity [47], and VEGF/VEGF receptor blockade has been shown to reduce myeloid-derived suppressor cells in tumors and blood in preclinical tumor models and human cancers [18]. A recent trial has shown the improved clinical outcome associated with atezolizumab (anti-PD-L1 antibody) and bevacizumab (anti-VEGFA antibody) compared with atezolizumab monotherapy in the immune suppressed myeloid cell high subgroup, suggesting that the addition of bevacizumab to atezolizumab may overcome myeloid-cell mediated resistance [48]. In our present study, however, these cells could not be analyzed due to the limited sample volume, these findings should be tested in the future studies.

A previous study reported that Treg cells express high levels of VEGFR2 and that RAM therapy could reduce the frequency of VEGFR2^+^ Treg cells, thus corroborating the results of our study [31]. Another study reported that peripheral Treg-cell numbers were diminished by bevacizumab (anti-VEGFA antibody)-containing therapies in patients with colorectal cancer [16], although TILs were only assessed by IHC. In addition to IHC, we employed flow cytometry for TIL analyses. Flow cytometry has several advantages to explore the detailed molecular expression in TILs, although investigating the location of TILs depends on IHC. By combining flow cytometry and IHC, we were able to address the entire landscape of immunological status in tumor microenvironment even with tiny biopsy samples. We investigated both PBMCs and TILs and determined that the reduction in eTreg cells was more profound in TILs than in PBMCs. The inconsistent result with the previous study [16] could possibly be attributed to our definition of a Treg cell; while CD25^+^FOXP3^+^CD4^+^ T cells were defined as “Treg cells” in the previous study, we defined these more precisely as “eTreg cells” (CD45RA^−^FOXP3^high^CD4^+^ T cell). These eTreg cells have a suppressive function, while non-Treg CD45RA^−^FOXP3^low^CD4^+^ cells, which produce inflammatory cytokines such as IFN-γ are included in the CD25^+^FOXP3^+^CD4^+^ T cell population. Thus, our reported “eTreg cell” frequency in PBMCs was lower than the previously reported “Treg cell” frequency in PBMCs.

## Conclusions

We observed a significant difference in immune profiles between PBMCs and TILs and determined that PD-L1 expression and CD8^+^ T-cell infiltration were increased following RAM-containing therapy in patients with advanced GC. Based on more detailed immune profiles, both TIL eTreg cells and PD-1 expression by CD8^+^ T cells were decreased by RAM-containing therapies, an effect not observed in PBMCs. Further, high eTreg frequencies in TILs correlate with favorable responses to RAM-containing therapies. Thus, our study is the first to utilize human clinical samples to highlight the significance of TIL analyses and to propose RAM as an immuno-modulator in combination with ICB.

## Additional files

Additional file 1: **Table S1.** Gene list of the Oncomine™ Cancer Research Panel (OCP143). (DOCX 18 kb)

Additional file 2: **Table S2.** Patient characteristics. (DOCX 19 kb)

Additional file 3: **Table S3.** Detailed clinical characteristics and immune cell data. (DOCX 21 kb)

Additional file 4: **Figure S1.** Genomic features of patients with GC who received RAM-containing therapies. (DOCX 137 kb)

Additional file 5: **Figure S2.** Difference in patient characteristics according to eTreg-cell frequency in TIL. (DOCX 129 kb)

Additional file 6: **Figure S3.** Kinetic changes of eTreg cells and PD-1 expression by CD8^+^ T cells according to therapies in TILs. (DOCX 242 kb)

Additional file 7: **Figure S4.** Kinetic changes of CD45RA^−^FOXP3^−^CD4^+^ T cells and eTreg cells in CD3^+^ T cells. (DOCX 137 kb)

Additional file 8: **Figure S5.** Kinetic changes of IC molecule expression by CD8^+^ T cells in both PBMCs and TILs. (DOCX 203 kb)

Additional file 9: **Figure S6.** Kinetic changes of IC molecule expression by CD45RA^−^FOXP3^−^CD4^+^ T cells in both PBMCs and TILs. (DOCX 208 kb)

Additional file 10: **Figure S7.** Kinetic changes of IC molecule expression by eTreg cells in both PBMCs and TILs. (DOCX 108 kb)

Additional file 11: **Figure S8.** Comparison of IC expression by eTreg cells between pre-and post-treatment in both PBMCs and TILs. (DOCX 240 kb)

Additional file 12: **Figure S9.** % of eTreg-cell reduction and % of PD-1 reduction on CD8^+^ T cells and clinical responses. (DOCX 77 kb)

Additional file 13: **Figure S10.** Impact of anti-VEGFR2 blockade on PBMCs in vitro. (DOCX 266 kb)

## Abbreviations

CPT-11: Irinotecan
eTreg cell: effector regulatory T cell
GC: Gastric cancer
HER2: Human epidermal growth factor receptor type 2
IC: Immune checkpoint
ICB: Immune checkpoint blockade
IHC: Immunohistochemistry
NSCLC: Non-small cell lung cancer
OS: Overall survival
PBMC: Peripheral blood mononuclear cell
PD: Progressive disease
PFS: Progression-free survival
PR: Partial response
PTX: Paclitaxel
RAM: Ramucirumab
SD: Stable disease
TIL: Tumor-infiltrating lymphocyte
Treg cell: Regulatory T cell
VEGF: Vascular endothelial growth factor
VEGFR2: Vascular endothelial growth factor receptor 2
