# Supplementary material for: Targeting VEGFR2 with Ramucirumab strongly impacts effector/ activated regulatory T cells and CD8+ T cells in the tumor microenvironment
Source: J Immunother Cancer. 2018 Oct 11;6:106. doi: 10.1186/s40425-018-0403-1 (PMC6186121; doi:10.1186/s40425-018-0403-1)
Supplement: Supplementary file 1 — Table S1. Gene list of the Oncomine™ Cancer Research Panel (OCP143). (DOCX 18 kb) [file 40425_2018_403_MOESM1_ESM.docx]

Table S1 Gene list of the Oncomine^TM^ Cancer Research Panel (OCP143)

| **Hotspot genes** | **Full-length genes** | **Copy number genes** | **Gene fusions (inter- and intragenic)** |
| --- | --- | --- | --- |
| *ABL1* | *APC* | *ACVRL1* | *ABL1* |
| *AKT1* | *ATM* | *AKT1* | *AKT3* |
| *ALK* | *BAP1* | *APEX1* | *ALK* |
| *AR* | *BRCA1* | *AR* | *AXL* |
| *ARAF* | *BRCA2* | *ATP11B* | *BRAF* |
| *BRAF* | *CDH1* | *BCL2L1* | *CDK4* |
| *BTK* | *CDKN2A* | *BCL9* | *ERBB2* |
| *CBL* | *FBXW7* | *BIRC2* | *ERG* |
| *CDK4* | *GATA3* | *BIRC3* | *ETV1* |
| *CHEK2* | *MSH2* | *CCND1* | *ETV4* |
| *CSF1R* | *NF1* | *CCNE1* | *ETV5* |
| *CTNNB1* | *NF2* | *CD274* | *FGFR1* |
| *DDR2* | *NOTCH1* | *CD44* | *FGFR2* |
| *EGFR* | *PIK3R1* | *CDK4* | *FGFR3* |
| *ERBB2* | *PTCH1* | *CDK6* | *NTRK1* |
| *ERBB3* | *PTEN* | *CSNK2A1* | *NTRK3* |
| *ERBB4* | *RB1* | *DCUN1D1* | *PDGFRA* |
| *ESR1* | *SMAD4* | *EGFR* | *PPARG* |
| *EZH2* | *SMARCB1* | *ERBB2* | *RAF1* |
| *FGFR1* | *STK11* | *FGFR1* | *RET* |
| *FGFR2* | *TET2* | *FGFR2* | *ROS1* |
| *FGFR3* | *TP53* | *FGFR3* |  |
| *FLT3* | *TSC1* | *FGFR4* |  |
| *FOXL2* | *TSC2* | *FLT3* |  |
| *GATA2* | *VHL* | *GAS6* |  |
| *GNA11* | *WT1* | *IGF1R* |  |
| *GNAQ* |  | *IL6* |  |
| *GNAS* |  | *KIT* |  |
| *HNF1A* |  | *KRAS* |  |
| *HRAS* |  | *KRAS* |  |
| *IDH1* |  | *MCL1* |  |
| *IDH2* |  | *MDM2* |  |
| *IFITM1* |  | *MDM4* |  |
| *IFITM3* |  | *MET* |  |
| *JAK1* |  | *MYC* |  |
| *JAK2* |  | *MYCL* |  |
| *JAK3* |  | *MYCN* |  |
| *KDR* |  | *MYO18A* |  |
| *KIT* |  | *NKX2-1* |  |
| *KNSTRN* |  | *NKX2-8* |  |
| *KRAS* |  | *PDCD1LG2* |  |
| *MAGOH* |  | *PDGFRA* |  |
| *MAP2K1* |  | *PIK3CA* |  |
| *MAP2K2* |  | *PNP* |  |
| *MAPK1* |  | *PPARG* |  |
| *MAX* |  | *RPS6KB1* |  |
| *MED12* |  | *SOX2* |  |
| *MET* |  | *TERT* |  |
| *MLH1* |  | *TIAF1* |  |
| *MPL* |  | *ZNF217* |  |
| *MTOR* |  |  |  |
| *MYD88* |  |  |  |
| *NFE2L2* |  |  |  |
| *NPM1* |  |  |  |
| *NRAS* |  |  |  |
| *PAX5* |  |  |  |
| *PDGFRA* |  |  |  |
| *PIK3CA* |  |  |  |
| *PPP2R1A* |  |  |  |
| *PTPN11* |  |  |  |
| *RAC1* |  |  |  |
| *RAF1* |  |  |  |
| *RET* |  |  |  |
| *RHEB* |  |  |  |
| *RHOA* |  |  |  |
| *SF3B1* |  |  |  |
| *SMO* |  |  |  |
| *SPOP* |  |  |  |
| *SRC* |  |  |  |
| *STAT3* |  |  |  |
| *U2AF1* |  |  |  |
| *XPO1* |  |  |  |
