# Supplementary material for: Targeting VEGFR2 with Ramucirumab strongly impacts effector/ activated regulatory T cells and CD8+ T cells in the tumor microenvironment
Source: J Immunother Cancer. 2018 Oct 11;6:106. doi: 10.1186/s40425-018-0403-1 (PMC6186121; doi:10.1186/s40425-018-0403-1)
Supplement: Supplementary file 2 — Table S2. Patient characteristics. (DOCX 19 kb) [file 40425_2018_403_MOESM2_ESM.docx]

**Table S2 Patient characteristics**

| Features | N (20) |
| --- | --- |
| **Age** | 46-79 years (mean, 66; median, 67) |
| **Sex**  Male  Female | 13  7 |
| **PS**  0-1  2-3 | 20  0 |
| **Histology**  Intestinal  Diffuse | 11  9 |
| ***ERBB2***  Not amplified  Amplified  Unknown | 15  2  3 |
| ***TP53***  Wild-type  Mutated  Unknown | 7  10  3 |
| **MMR status**  Proficient  Deficient | 20  0 |
| **EBV infection**  No  Yes | 19  1 |
| **Treatment line**  2^nd^ line  3^rd^ line- | 14  6 |
| **Regimen**  RAM monotherapy  RAM+PTX or nabPTX  RAM+CPT-11 | 3  12  5 |
| **Response**  PR  SD  PD | 6  11  3 |
| **Biopsy times** | 2-6 (mean, 3.25; median, 3) |
| **Median PFS** | 110 days |
| **Median OS** | 324 days |

RAM, ramucirumab; PTX, paclitaxel; CPT-11, irinotecan; PFS, progression-free survival; OS, overall survival
