# Supplementary material for: Targeting VEGFR2 with Ramucirumab strongly impacts effector/ activated regulatory T cells and CD8+ T cells in the tumor microenvironment
Source: J Immunother Cancer. 2018 Oct 11;6:106. doi: 10.1186/s40425-018-0403-1 (PMC6186121; doi:10.1186/s40425-018-0403-1)
Supplement: Supplementary file 3 — Table S3. Detailed clinical characteristics and immune cell data. (DOCX 21 kb) [file 40425_2018_403_MOESM3_ESM.docx]

Table S3 Detailed clinical characteristics and immune cell data

| **ID** | **Age and sex** | **Histology** | **Treatment line** | **Regimen** | **Response** | **RTK/RAS/PI3K** | ***TP53*** | **MMR status** | **EBV** | **PD-L1 IHC (TC)** | **PD-L1 IHC (IC)** | **CD8 IHC** | **eTreg cells in CD4^+^TILs** | **PD-1 on CD8^+^ TILs** |
| --- | --- | --- | --- | --- | --- | --- | --- | --- | --- | --- | --- | --- | --- | --- |
| 001 | 73, F | Diffuse | 3 | RAM | SD | NA | NA | Proficient | (-) | (-)→(-) | (-)→(+) | ↑ | ↓ | ↓↓ |
| 002 | 67, F | Intestinal | 2 | RAM+nabPTX | PR | *STK11* | Wild | Proficient | (-) | NA | NA | NA | ↓ | ↓↓ |
| 003 | 46, M | Diffuse | 2 | RAM+nabPTX | PR | NA | NA | Proficient | (-) | NA | NA | NA | ↓↓ | ↓ |
| 004 | 67, M | Diffuse | 2 | RAM+PTX | SD | *PIK3CA* | Wild | Proficient | (-) | (-)→(+) | (+)→(+) | → | ↓ | ↓↓ |
| 007 | 64, M | Intestinal | 2 | RAM+PTX | PR | - | Wild | Proficient | (-) | (-)→(-) | (+)→(+) | → | NA | NA |
| 008 | 54, M | Diffuse | 4 | RAM+PTX | PD | *MET* | Wild | Proficient | (+) | NA | NA | NA | ↓ | ↓↓ |
| 010 | 67, F | Intestinal | 3 | RAM+PTX | SD | *KRAS* | Wild | Proficient | (-) | (-)→(-) | (+)→(+) | → | ↑↑ | ↓↓ |
| 011 | 73, F | Intestinal | 2 | RAM | SD | - | Mutated | Proficient | (-) | NA | NA | NA | ↓ | ↓↓ |
| 012 | 79, F | Intestinal | 5 | RAM+CPT11 | PR | *ERBB2* | Mutated | Proficient | (-) | (-)→(-) | (-)→(+) | ↑ | ↓↓ | ↓ |
| 013 | 74, M | Diffuse | 3 | RAM+PTX | PR | *ERBB2, MAP2K1* | Wild | Proficient | (-) | NA | NA | NA | ↓ | ↓↓ |
| 015 | 51, F | Intestinal | 2 | RAM+PTX | SD | NA | NA | Proficient | (-) | (-)→(-) | (+)→(+) | → | ↓↓ | ↓↓ |
| 016 | 73, M | Intestinal | 2 | RAM | SD | *FGFR2* | Mutated | Proficient | (-) | NA | NA | NA | ↑ | ↑↑ |
| 018 | 60, M | Intestinal | 2 | RAM+PTX | SD | - | Mutated | Proficient | (-) | NA | NA | NA | ↓ | ↓ |
| 019 | 66, M | Intestinal | 2 | RAM+PTX | PD | *ERBB2* | Mutated | Proficient | (-) | NA | NA | NA | NA | NA |
| 020 | 76, M | Diffuse | 2 | RAM+PTX | SD | *MET, KRAS* | Mutated | Proficient | (-) | (-)→(-) | (+)→(+) | ↑ | ↓ | ↓ |
| 024 | 71, M | Diffuse | 2 | RAM+PTX | SD | - | Mutated | Proficient | (-) | NA | NA | NA | → | ↑ |
| 025 | 68, M | Intestinal | 4 | RAM+CPT11 | PD | - | Mutated | Proficient | (-) | NA | NA | NA | ↓ | ↓↓ |
| 026 | 55, M | Intestinal | 2 | RAM+CPT11 | PR | *STK11* | Mutated | Proficient | (-) | NA | NA | NA | ↓ | ↓↓ |
| 028 | 63, M | Diffuse | 2 | RAM+CPT11 | PD | *EGFR, RHOA* | Wild | Proficient | (-) | NA | NA | NA | ↓ | ↓↓ |
| 033 | 72, F | Diffuse | 2 | RAM+CPT11 | SD | *MET* | Mutated | Proficient | (-) | NA | NA | NA | → | ↓ |

M, male; F. female; RAM, ramucirumab; PTX, paclitaxel; CPT11, irinotecan; PR, partial response; SD, stable disease; PD, progressive disease; RTK, receptor tyrosine kinase; MMR, mismatch repair; EBV, Epstein-Barr virus, TC, tumor cell; IC, immune cell; IHC, immunohistochemistry; TIL, tumor-infiltrating lymphocyte; NA, not available.
