# Supplementary material for: Targeting VEGFR2 with Ramucirumab strongly impacts effector/ activated regulatory T cells and CD8+ T cells in the tumor microenvironment
Source: J Immunother Cancer. 2018 Oct 11;6:106. doi: 10.1186/s40425-018-0403-1 (PMC6186121; doi:10.1186/s40425-018-0403-1)
Supplement: Supplementary file 4 — Figure S1. Genomic features of patients with GC who received RAM-containing therapies. (DOCX 137 kb) [file 40425_2018_403_MOESM4_ESM.docx]

Figure S1 Genomic features of patients with GC who received RAM-containing therapies.


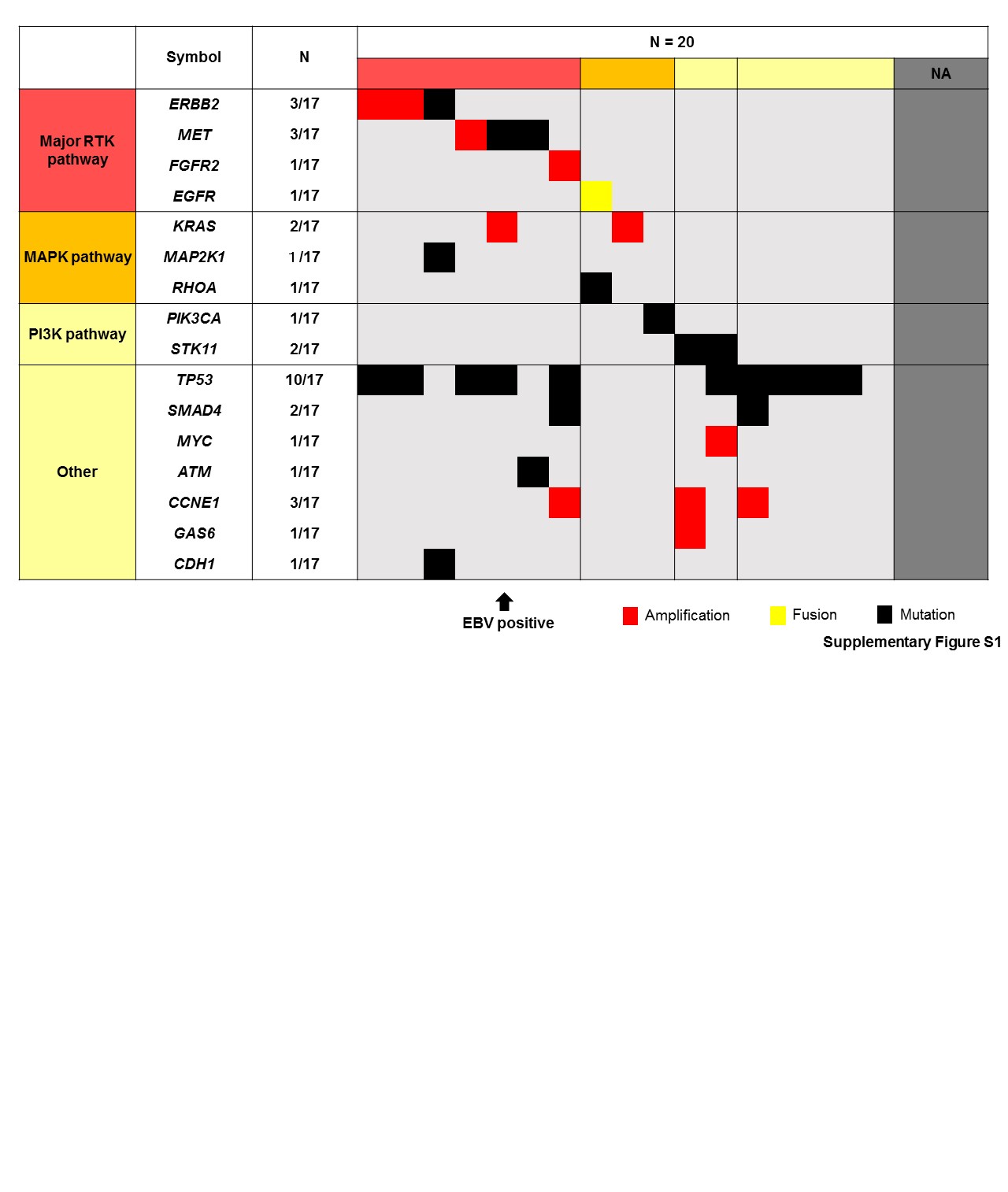
DNA and RNA were extracted from FFPE tumor samples before treatment and were subjected to the Oncomine™ Comprehensive Assay version 3 to detect gene mutations, copy number variants and fusions across multiple genes. *TP53* were frequently mutated (10/17) and *ERBB2*, *MET*, *FGFR2*, or *KRAS*, amplification, or a *RHOA* mutation were also identified. In contrast, all were MMR proficient GC and only one was EBV-positive GC (an arrow). RTK, receptor tyrosine kinase; NA, not available.
