# Supplementary material for: Targeting VEGFR2 with Ramucirumab strongly impacts effector/ activated regulatory T cells and CD8+ T cells in the tumor microenvironment
Source: J Immunother Cancer. 2018 Oct 11;6:106. doi: 10.1186/s40425-018-0403-1 (PMC6186121; doi:10.1186/s40425-018-0403-1)
Supplement: Supplementary file 5 — Figure S2. Difference in patient characteristics according to eTreg-cell frequency in TIL. (DOCX 129 kb) [file 40425_2018_403_MOESM5_ESM.docx]

**Figure S2 Difference in patient characteristics according to eTreg-cell frequency in TIL.**


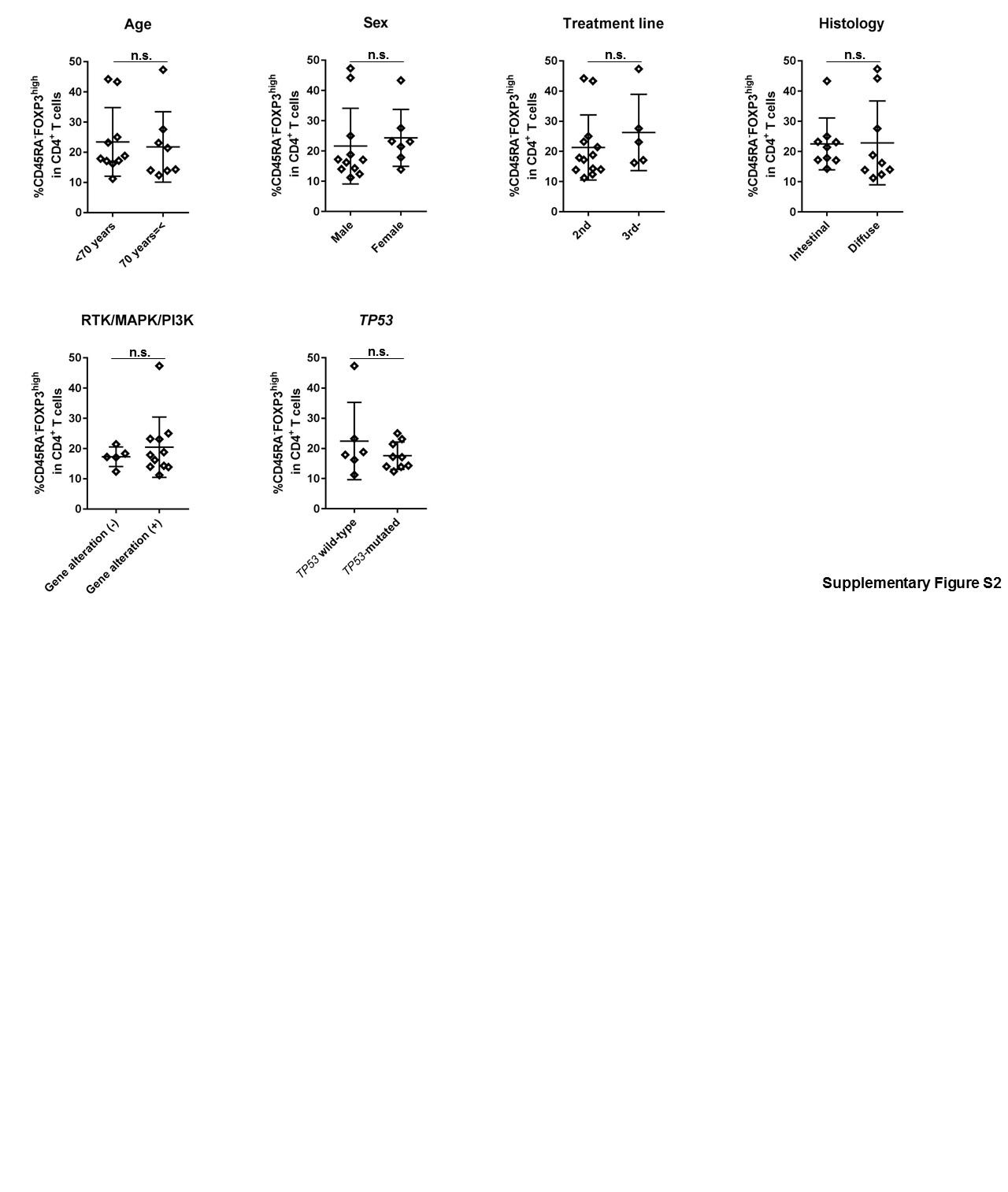


TILs were collected and were subjected to flow cytometry to analyze immune profiles in detail. FOXP3^+^CD4^+^ T cells were investigated based on expression levels of both FOXP3 and the naïve marker CD45RA. No correlations between eTreg-cell frequency and patients’ characteristics, including age, sex, histology, treatment line, receptor tyrosine kinase (RTK), MAPK, or PI3K gene alteration, and *TP53* status was observed.
