# Supplementary material for: Targeting VEGFR2 with Ramucirumab strongly impacts effector/ activated regulatory T cells and CD8+ T cells in the tumor microenvironment
Source: J Immunother Cancer. 2018 Oct 11;6:106. doi: 10.1186/s40425-018-0403-1 (PMC6186121; doi:10.1186/s40425-018-0403-1)
Supplement: Supplementary file 6 — Figure S3. Kinetic changes of eTreg cells and PD-1 expression by CD8+ T cells according to therapies in TILs. (DOCX 242 kb) [file 40425_2018_403_MOESM6_ESM.docx]

**Figure S3 Kinetic changes of eTreg cells and PD-1 expression by CD8^+^ T cells according to therapies in TILs.**

**
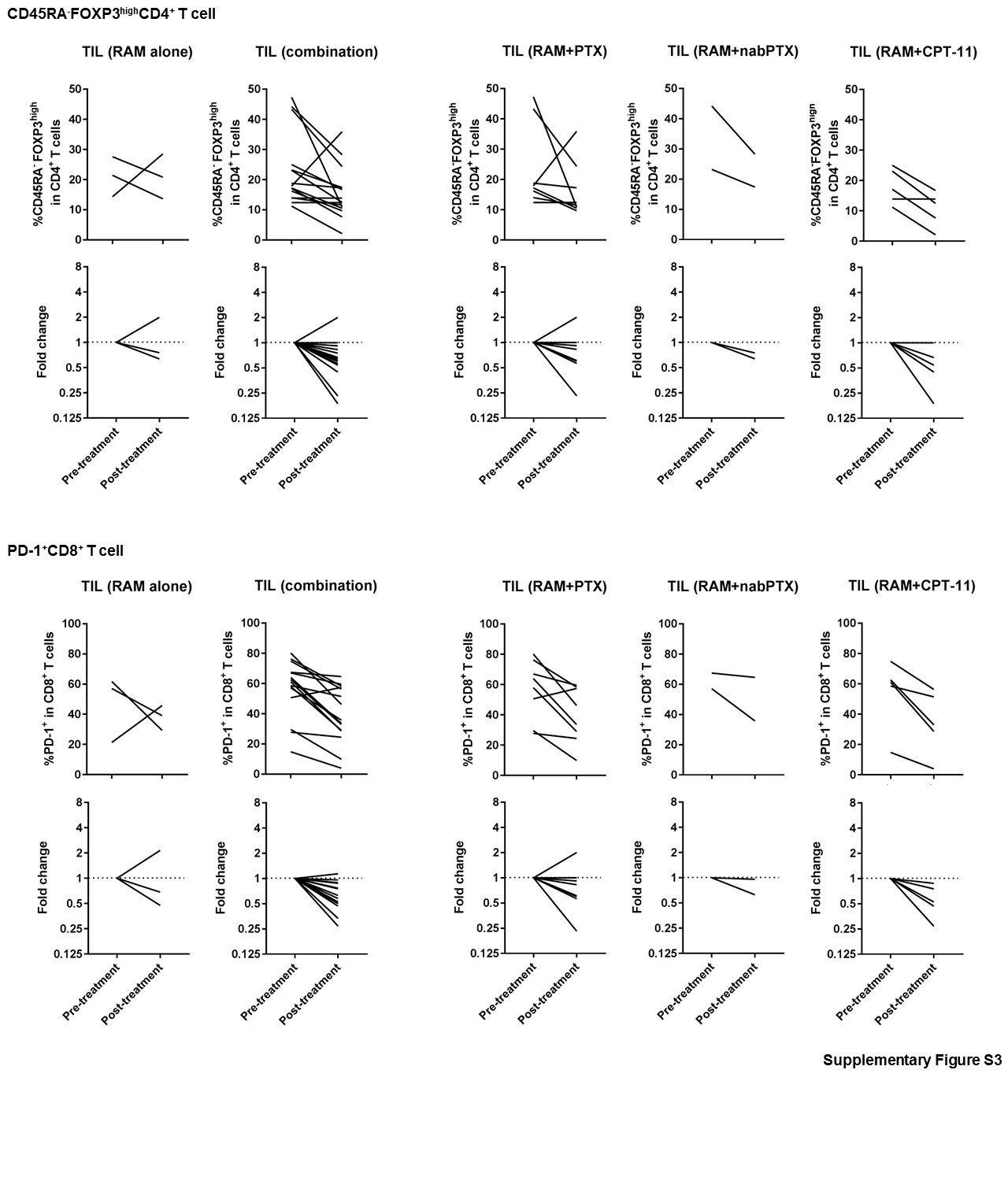
**

Pre- and post-treatment TILs were collected and were subjected to flow cytometry to analyze immune profiles in detail. The tendencies of reduction were observed in both RAM monotherapy and RAM with chemotherapy including RAM+PTX, RAM+nabPTX, or RAM+CPT-11.
