# Supplementary material for: Targeting VEGFR2 with Ramucirumab strongly impacts effector/ activated regulatory T cells and CD8+ T cells in the tumor microenvironment
Source: J Immunother Cancer. 2018 Oct 11;6:106. doi: 10.1186/s40425-018-0403-1 (PMC6186121; doi:10.1186/s40425-018-0403-1)
Supplement: Supplementary file 7 — Figure S4. Kinetic changes of CD45RA−FOXP3−CD4+ T cells and eTreg cells in CD3+ T cells. (DOCX 137 kb) [file 40425_2018_403_MOESM7_ESM.docx]

Figure S4 Kinetic changes of CD45RA^-^FOXP3^-^CD4^+^ T cells and eTreg cells in CD3^+^ T cells.

**
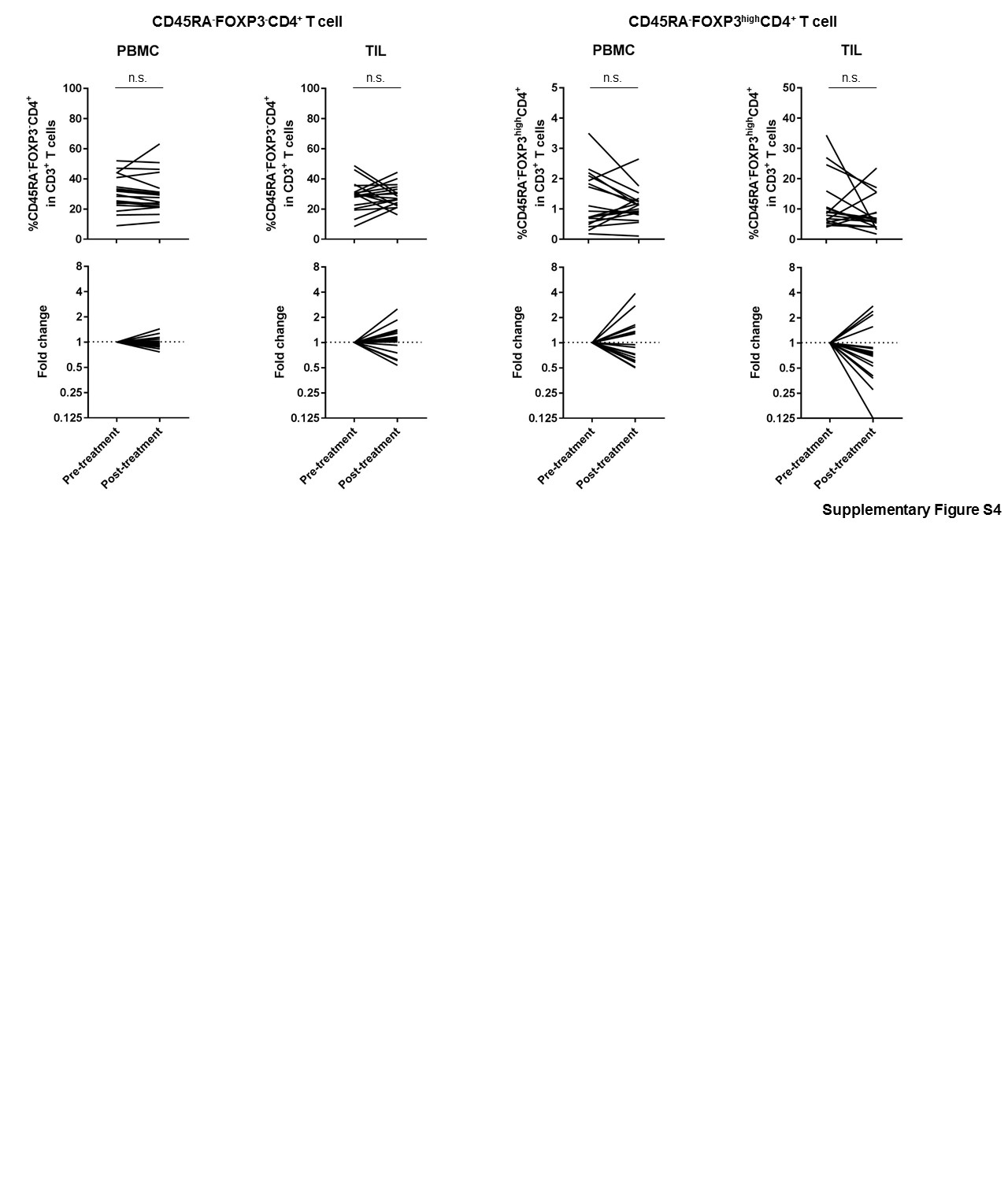
**

Pre- and post-treatment TILs were collected and were subjected to flow cytometry to analyze immune profiles in detail. The tendency of reduction of eTreg cells in CD3^+^ TILs were observed, whereas the change of CD45RA^-^FOXP3^-^CD4^+^ T cells in CD3^+^ TILs was comparable.
