# Supplementary material for: Targeting VEGFR2 with Ramucirumab strongly impacts effector/ activated regulatory T cells and CD8+ T cells in the tumor microenvironment
Source: J Immunother Cancer. 2018 Oct 11;6:106. doi: 10.1186/s40425-018-0403-1 (PMC6186121; doi:10.1186/s40425-018-0403-1)
Supplement: Supplementary file 8 — Figure S5. Kinetic changes of IC molecule expression by CD8+ T cells in both PBMCs and TILs. (DOCX 203 kb) [file 40425_2018_403_MOESM8_ESM.docx]

Figure S5 Kinetic changes of IC molecule expression by CD8^+^ T cells in both PBMCs and TILs.


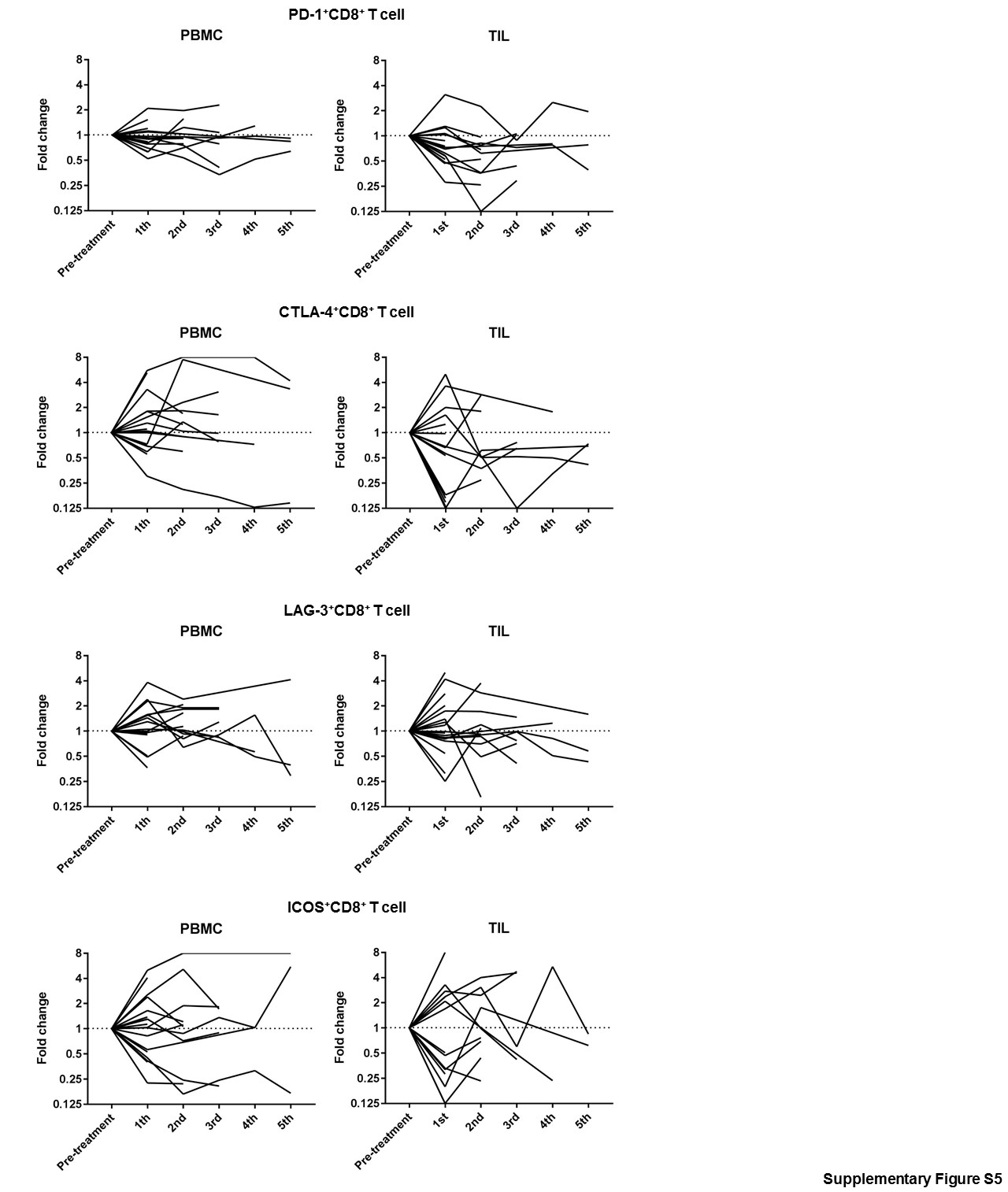
 Pre- and post-treatment TILs and PBMCs were collected and were subjected to flow cytometry to analyze immune profiles in detail. Kinetic changes of IC molecule expression by CD8^+^ T cells exhibited dynamic changes.
