# Supplementary material for: Targeting VEGFR2 with Ramucirumab strongly impacts effector/ activated regulatory T cells and CD8+ T cells in the tumor microenvironment
Source: J Immunother Cancer. 2018 Oct 11;6:106. doi: 10.1186/s40425-018-0403-1 (PMC6186121; doi:10.1186/s40425-018-0403-1)
Supplement: Supplementary file 11 — Figure S8. Comparison of IC expression by eTreg cells between pre-and post-treatment in both PBMCs and TILs. (DOCX 240 kb) [file 40425_2018_403_MOESM11_ESM.docx]

Figure S8 Comparison of IC expression by eTreg cells between pre-and post-treatment in both PBMCs and TILs


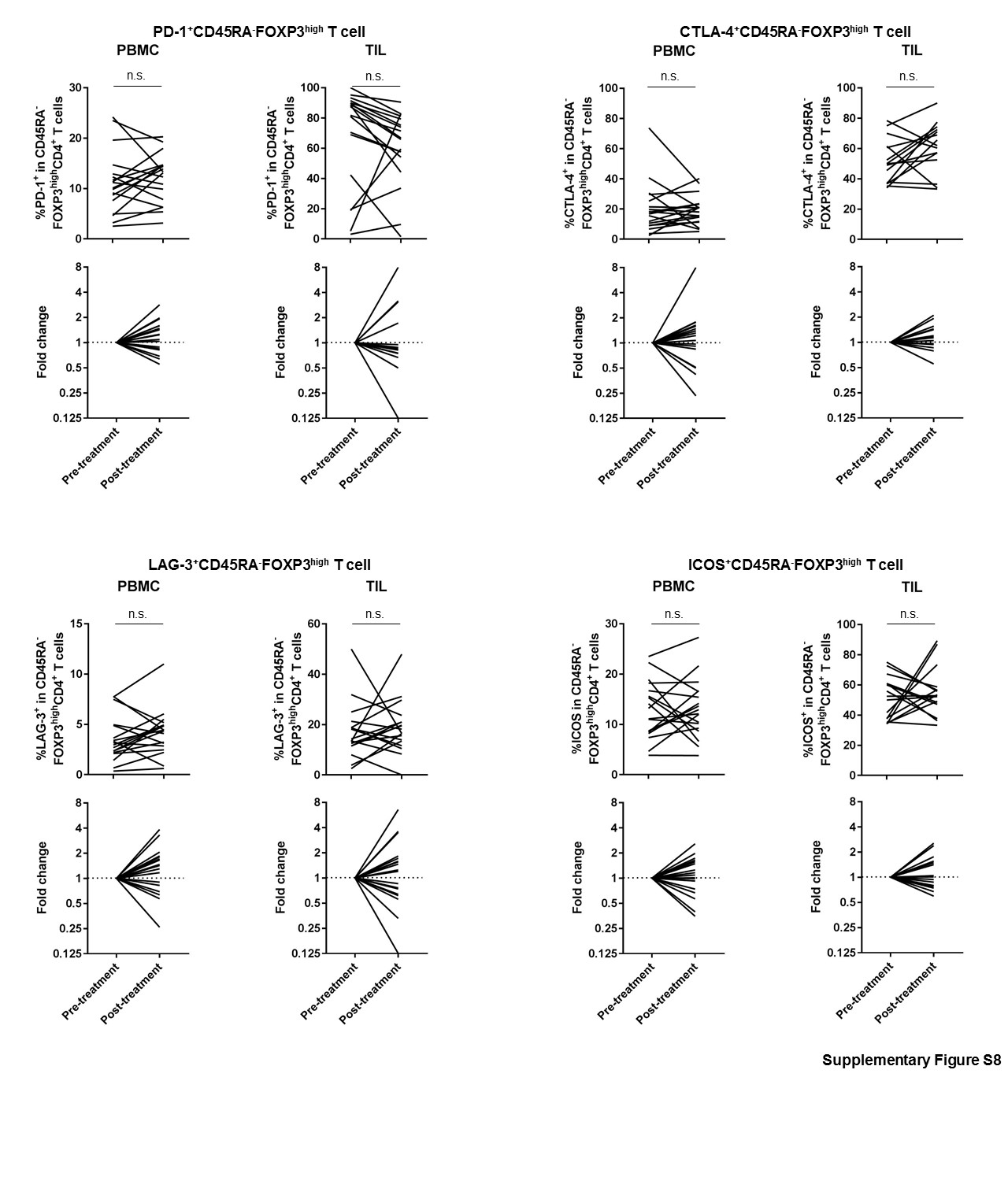
Pre- and post-treatment TILs and PBMCs were collected and were subjected to flow cytometry to analyze immune profiles in detail. There was no significant difference of IC expression including PD-1, CTLA-4, LAG-3, and ICOS in both PBMCs and TILs between pre- and post-treatment.
