# Supplementary material for: Targeting VEGFR2 with Ramucirumab strongly impacts effector/ activated regulatory T cells and CD8+ T cells in the tumor microenvironment
Source: J Immunother Cancer. 2018 Oct 11;6:106. doi: 10.1186/s40425-018-0403-1 (PMC6186121; doi:10.1186/s40425-018-0403-1)
Supplement: Supplementary file 12 — Figure S9. % of eTreg-cell reduction and % of PD-1 reduction on CD8+ T cells and clinical responses. (DOCX 77 kb) [file 40425_2018_403_MOESM12_ESM.docx]

**Figure S9 % of eTreg-cell reduction and % of PD-1 reduction on CD8^+^ T cells and clinical responses.**


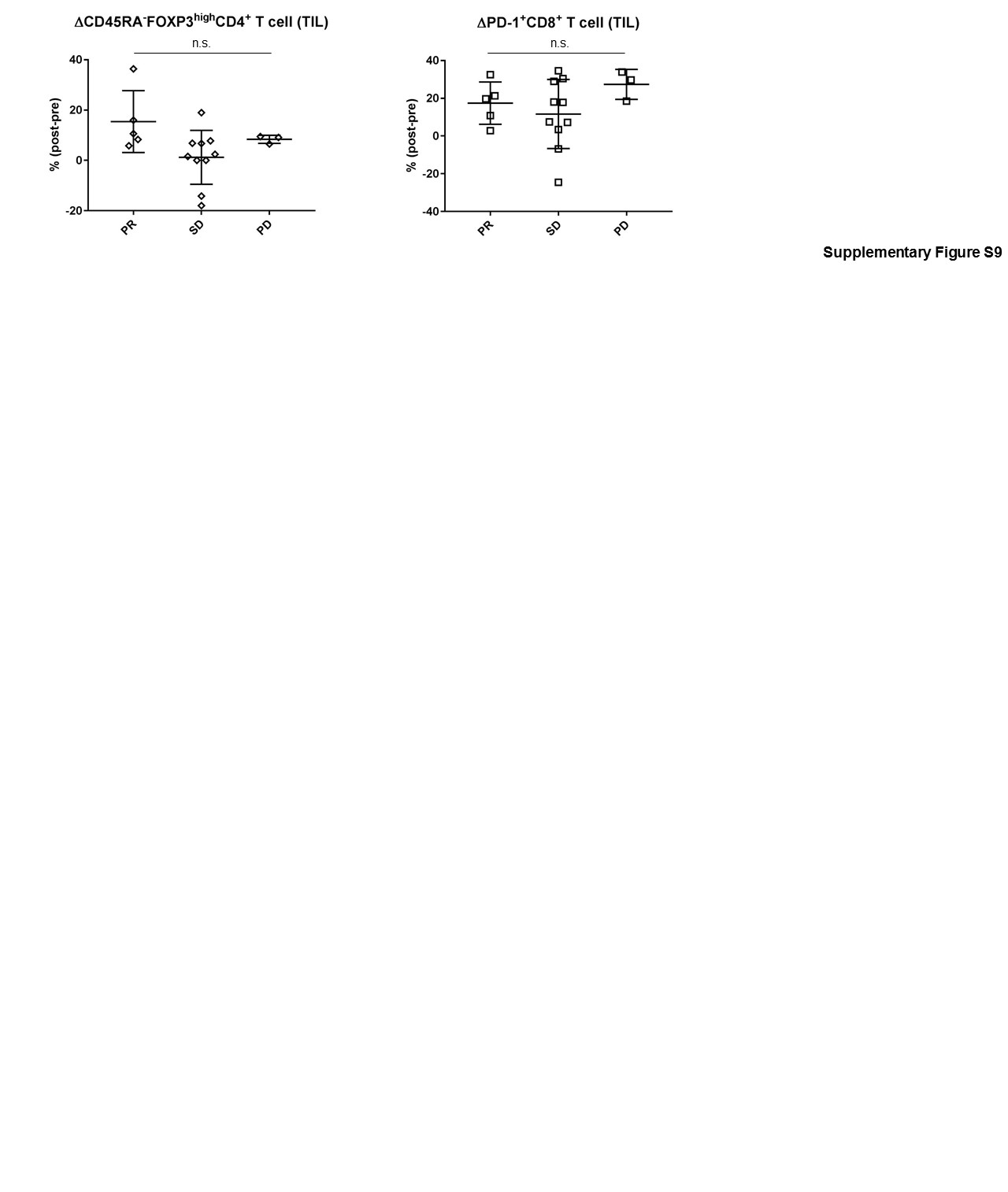
 Pre- and post-treatment TILs were collected and were subjected to flow cytometry to analyze immune profiles in detail to investigate kinetic changes during RAM-containing therapy. There was no significant difference in % of eTreg-cell reduction and % of PD-1 reduction on CD8^+^ T cells among patinets with PR, SD, and PD. But, patients with PR tended to have more % of eTreg-cell reduction.
