# Supplementary material for: Targeting VEGFR2 with Ramucirumab strongly impacts effector/ activated regulatory T cells and CD8+ T cells in the tumor microenvironment
Source: J Immunother Cancer. 2018 Oct 11;6:106. doi: 10.1186/s40425-018-0403-1 (PMC6186121; doi:10.1186/s40425-018-0403-1)
Supplement: Supplementary file 13 — Figure S10. Impact of anti-VEGFR2 blockade on PBMCs in vitro. (DOCX 266 kb) [file 40425_2018_403_MOESM13_ESM.docx]

**Figure S10 Impact of anti-VEGFR2 blockade on PBMCs *in vitro*.**

**
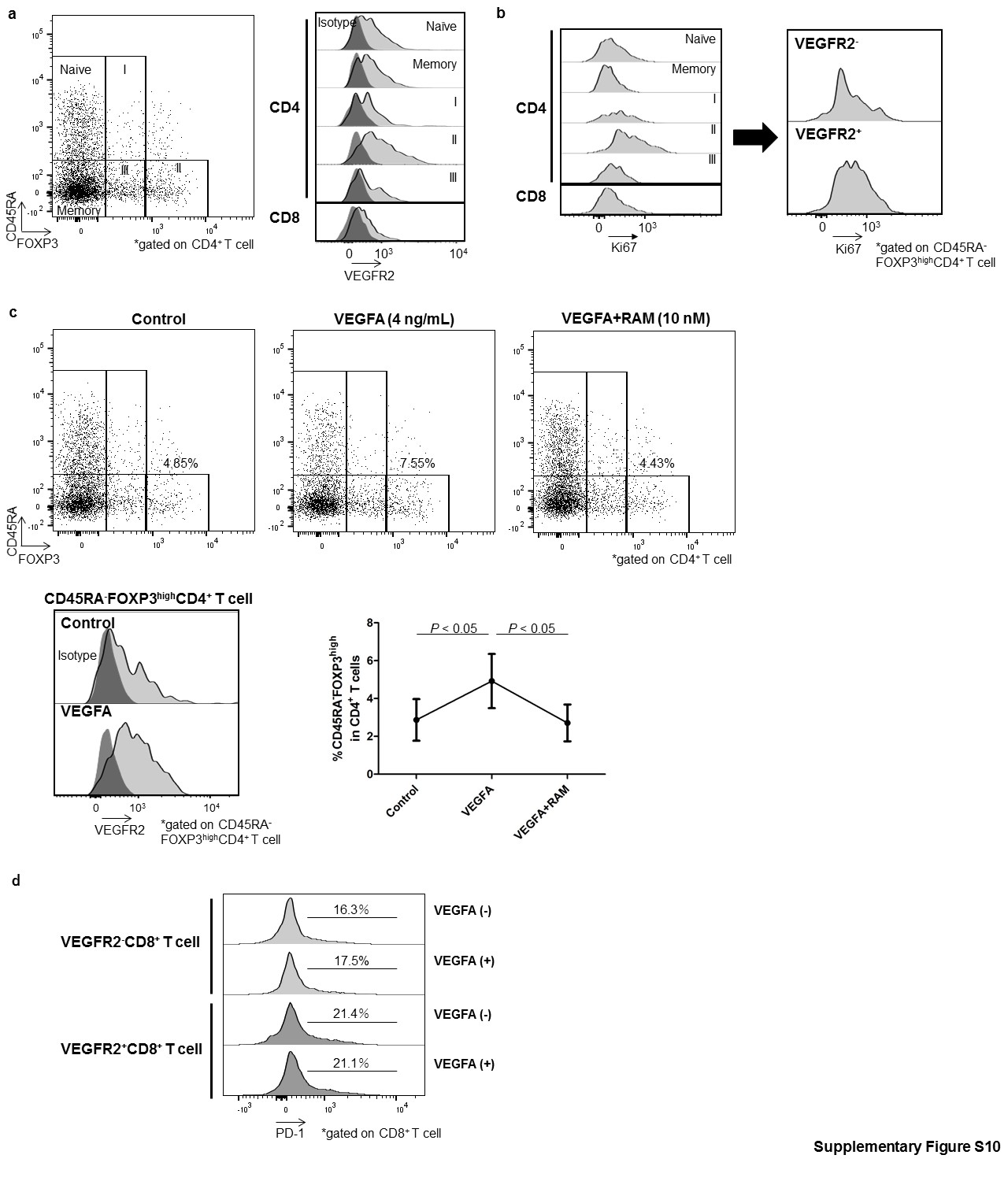
 (a)** VEGFR2 expression in several T-cell subsets. The highest expression of VEGFR2 was observed in CD45RA^−^FOXP3^high^CD4^+^ T cells (Fraction II; eTreg cells). **(b)** Proliferation of several T-cell subsets. Proliferation was evaluated using Ki67 staining. eTreg cells expressed high levels of Ki67. VEGFR2^+^ eTreg cells had higher expression levels of Ki67 compared to VEGFR2^-^ eTreg cells. **(c)** Kinetic changes in eTreg cells treated with or without VEGFA and/or RAM. When PBMCs were treated with the VEGFR2 ligand VEGFA (4 ng/mL), eTreg cell numbers increased, especially VEFR2^+^ eTreg cells. RAM reversed the VEGFA-induced increase in eTreg cell numbers. Experiments were performed using PBMCs from 3 different healthy donors. **(d)** Kinetic changes of PD-1^+^CD8^+^ T cells treated with or without VEGFA. VEGFR2^+^CD8^+^ T cells exhibited a little higher PD-1 expression compared with VEGFR2^-^CD8^+^ T cells, while PD-1 expression by CD8^+^ T cells did not increase after VEGFA treatment.
